# Identification of the Genes Related to the Glycogen Metabolism in Hyperthermophilic Archaeon, *Sulfolobus acidocaldarius*

**DOI:** 10.3389/fmicb.2021.661053

**Published:** 2021-05-13

**Authors:** Areum Lee, Eunji Bae, Jihee Park, Kyoung-Hwa Choi, Jaeho Cha

**Affiliations:** ^1^Department of Integrated Biological Science, Pusan National University, Busan, South Korea; ^2^Research Development Institute, Cowellmedi, Busan, South Korea; ^3^Department of Southern Area Crop Science, Upland Crop Breeding Research Division, National Institute of Crop Science, Rural Development Administration, Miryang, South Korea; ^4^Department of Microbiology, Pusan National University, Busan, South Korea

**Keywords:** thermophiles, *Sulfolobus acidocaldarius*, glycogen synthesis, glycogen degradation, role of glycogen

## Abstract

Glycogen is a polysaccharide that comprises α-1,4-linked glucose backbone and α-1,6-linked glucose polymers at the branching points. It is widely found in organisms ranging from bacteria to eukaryotes. The physiological role of glycogen is not confined to being an energy reservoir and carbon source but varies depending on organisms. *Sulfolobus acidocaldarius*, a thermoacidophilic archaeon, was observed to accumulate granular glycogen in the cell. However, the role of glycogen and genes that are responsible for glycogen metabolism in *S. acidocaldarius* has not been identified clearly. The objective of this study is to identify the gene cluster, which is composed of enzymes that are predicted to be involved in the glycogen metabolism, and confirm the role of each of these genes by constructing deletion mutants. This study also compares the glycogen content of mutant and wild type and elucidates the role of glycogen in this archaeon. The glycogen content of *S. acidocaldarius* MR31, which is used as a parent strain for constructing the deletion mutant in this study, was increased in the early and middle exponential growth phases and decreased during the late exponential and stationary growth phases. The pattern of the accumulated glycogen was independent to the type of supplemented sugar. In the comparison of the glycogen content between the gene deletion mutant and MR31, glycogen synthase (GlgA) and α-amylase (AmyA) were shown to be responsible for the synthesis of glycogen, whereas glycogen debranching enzyme (GlgX) and glucoamylase (Gaa) appeared to affect the degradation of glycogen. The expressions of *glgC–gaa–glgX* and *amyA–glgA* were detected by the promoter assay. This result suggests that the gradual decrease of glycogen content in the late exponential and stationary phases occurs due to the increase in the gene expression of *glgC–gaa–glgX*. When the death rate in nutrient limited condition was compared among the wild type strain, the glycogen deficient strain and the strain with increased glycogen content, the death rate of the glycogen deficient strain was found to be higher than any other strain, thereby suggesting that the glycogen in *S. acidocaldarius* supports cell maintenance in harsh conditions.

## Introduction

Many organisms ranging from bacteria to eukaryotes accumulate carbon and energy in a specific form as reservoirs to prepare for harsh conditions such as temporary starvation (Preiss et al., 1975; Preiss, 1984). The most common form of energy and carbon storage can be divided into two groups: polysaccharides such as glycogen and starch, and lipids such as poly-β-hydroxybutyrate (Wilkinson, 1959, 1963). Although the starch is only found in photosynthetic eukaryotes and their derivatives, the glycogen is found in a majority of organisms of all three domains (Ball and Morell, 2003).

The role of glycogen is not limited to the storage of energy and carbon source but is also extended to various criteria (Preiss, 1984; Preiss and Romeo, 1990; Iglesias and Preiss, 1992). In *Corynebacterium glutamicum*, the accumulated glycogen is used as a substrate for producing trehalose, which is a compatible solute in responding to osmotic stress (Seibold and Eikmanns, 2007). The glycogen plays an important role in the differentiation and sporulation of *Bacillus subtilis* and *Myxococcus xanthus*, respectively (Kiel et al., 1994; Nariya and Inouye, 2003). In *M. xanthus*, the glycogen accumulated during the stationary phase plays an important role in sporulation. The *pfk–pkn4* deletion strain of *M. xanthus*, which cannot utilize accumulated glycogen during the developmental stage, showed a poor spore yield as compared to the wild type strain. In *Streptomyces coelicolor* A3(2), the accumulation of the glycogen was observed during two distinctive differentiation phases. The result revealed that GlgC1 was responsible for the formation of glycogen accumulated in Phase 1, thereby indicating that glycogen plays an active role in the differentiation of *S. coelicolor* A3(2) (Plaskitt and Chater, 1995; Martin et al., 1997; Nariya and Inouye, 2003). In *Escherichia coli*, glycogen metabolism is controlled by the nutritional and energy status, and plays a crucial role in cellular processes, ranging from energy production to cell-to-cell communication (Nariya and Inouye, 2003; Eydallin et al., 2007). A study also found that in *Mycobacterium smegmatis*, the glycogen plays a role as a carbon capacitor for regulating downstream carbon and energy fluxes (Belanger and Hatfull, 1999). Glycogen is continuously accumulated during the cell growth and degraded at the exponential growth phase by the reaction of GlgE. The *glgE* deletion mutant of *M. smegmatis* showed an abnormal growth rate and colony morphology when permissive conditions are provided (Belanger and Hatfull, 1999).

The glycogen consists of α-1,4-linked glucose backbone and α-1,6-linked glucose polymers at the branching points. The ratio of α-1,6-linkage varies in all the organisms and at different stages of the glycogen synthesis (Iglesias and Preiss, 1992). The formation of the glycogen starts with the synthesis of ADP-glucose and pyrophosphate from the glucose-1-phosphate and ATP by the reaction of ADP-glucose phosphorylase (GlgC) (Ballicora et al., 2003). The α-1,4-glycosidic bond, which is a main structure of glycogen, is lengthened by the transportation of glucose from the ADP-glucose to the linear α-1,4-glucan as the reaction of glycogen synthase (GlgA) (Sheng et al., 2009). The branches are the result of the reaction of glycogen-branching enzyme (GlgB), which cuts a part of α-1,4-glucan and attaches to the remaining chain with α-1,6-linkages (Boyer and Preiss, 1977). The accumulated glycogen is then degraded by glycogen phosphorylase (GlgP), which cut glycogen from the non-reducing end and form glucose-1-phosphate and α-1,6-branched dextrin (Alonso-Casajús et al., 2006). Glycogen debranching enzyme (GlgX), which exerts activity toward the phosphorylase-limited dextrin, cuts the α-1,6-branch residues (Dauvillée et al., 2005).

Hyperthermophiles, which can grow optimally at temperature ranging from 80 to 110°C, are considered as the most ancient type of living cells because they are phylogenetically closer to the common ancestor. *Sulfolobus acidocaldarius*, a thermoacidophilic archaeon isolated from hot springs in Yellowstone National Park, is used as a model organism to investigate the cellular process including central dogma, DNA repair, and cell cycle (Schönheit and Schäfer, 1995). *S. acidocaldarius* is also considered as an interesting organism because it harbors enzymes that are related to various sugar metabolisms (Chen et al., 2005). Previous research found that *S. acidocaldarius* accumulates granular glycogen in cytosol (König et al., 1982), hence suggesting that this archaeon harbors an active system involved in the glycogen metabolism.

In the previous study, a glycogen-bound enzyme of *S. acidocaldarius* firstly known as polyphosphate kinase was identified as a glycogen synthase, GlgA (König et al., 1982; Skorko et al., 1989; Cardona et al., 2001). The three-dimensional structure of an archaeal glycogen debranching enzyme, TreX, of *S. solfataricus* shows similarity to the structure of GlgX. However, TreX has both transferase and glucosidase activity (Park et al., 2007; Woo et al., 2008). TreX activity was also observed in *S. shibatae* (Van et al., 2007). *S. solfataricus* harbors four putative glucoamylases (Sso2742, Sso2754, Sso0990, and Sso2473), but only Sso0990 showed the activity which hydrolyzes both α-1,4- and α-1,6-glycosidic linkages (Kim et al., 2004). Glucoamylase (Gaa) also found in *S. tokodaii* and its enzymatic property was characterized (Njoroge et al., 2005). GlgA, TreX, and Gaa were identified and characterized in *Sulfolobus* species but previous studies focused on the enzyme characterization, not the role of enzyme in the glycogen metabolism. Uncovering the physiological role of glycogen found in *S. acidocaldarius* and the system responsible for the glycogen metabolism in this strain could serve as a foundation to understand the role and mechanism of glycogen utilization in the organisms’ habitation in extreme conditions.

The objective of this study is to measure the intracellular glycogen content in *S. acidocaldarius* and identify the gene cluster, which encodes the enzymes that are expected to be related to glycogen metabolism by genetic approaches. Using the genetic disruption mutants, this study also aims to confirm the role of each gene within the gene cluster in the glycogen metabolism. Furthermore, this study investigates the role of glycogen in *S. acidocaldarius* based on the comparison of the death rates of the *S. acidocaldarius* MR31 and the glycogen deficient mutants.

## Materials and Methods

### Strains and Growth Conditions

The *S. acidocaldarius* strain MR31 (Reilly and Grogan, 2001), an uracil auxotroph with partial deletion on the gene encoding orotate phosphoribosyltransferase (*pyrE*) of *S. acidocaldarius* DSM 639, was used as a wild type in this study. *S. acidocaldarius* MR31 and the other strains were grown aerobically in the YT medium at 77°C, which is made from the Brock’s basal medium (Brock et al., 1972) with slight modifications. The medium was supplemented with 0.01% (w/v) of tryptone, 0.005% (w/v) of yeast extract. For the growth of *S. acidocaldarius* MR31 and Δ*amyA* strain, 20 μg/ml of uracil was supplemented. The pH was adjusted to 3.3 with 6 M H_2_SO_4_. In total, 0.2% (w/v) of glucose or other sugars were added as a carbon source. The growth was monitored at an optical density (OD) of 600 nm using spectrophotometer. The *Escherichia coli* strain DH5α used for cloning was grown at 37°C in the LB medium and ampicillin (100 μg/ml) or other antibiotics were added if necessary. The strains and plasmids used in this study were listed in Table 1.

**TABLE 1:** Strains and plasmids used in this study.

Strain and plasmid	Genetic marker and characteristics	References
Strains		
*S. acidocaldarius* MR31	*S. acidocaldarius* DSM 639 derivative, uracil auxotrophic mutant, Δ*pyrE*	Reilly and Grogan, 2001
Δ*glg*	*S. acidocaldarius* MR31 derivative, replacement of 8,596 bp within *glg* operon (*saci_11970*-*saci_1201*, *glgX*-*glgA*) with pyre gene (797 bp) from *S. solfataricus* P2	In this study
Δ*glgA*	*S. acidocaldarius* MR31 derivative, replacement of 1,525 bp within *saci_1201* operon with pyre gene (797 bp) from *S. solfataricus* P2	In this study
Δ*amyA*	*S. acidocaldarius* MR31 derivative, deletion of 830 bp within *saci_1200*	In this study
Δ*amyA*/*amyA*	Δ*amyA* derivative, transformation of pC::*amyA*.	In this study
Δ*glgP*	*S. acidocaldarius* MR31 derivative, replacement of 1,265 bp within *saci_0294* operon with pyre gene (797 bp) from *S. solfataricus* P2	In this study
Δ*glgX*	*S. acidocaldarius* MR31 derivative, replacement of 1,664 bp within *saci_1197* operon with pyre gene (797 bp) from *S. solfataricus* P2	In this study
Δ*gaa*	*S. acidocaldarius* MR31 derivative, replacement of 1,654 bp within *saci_1198* operon with pyre gene (797 bp) from *S. solfataricus* P2	In this study
P*_*glgP*_*-LacS	*S. acidocaldarius* MR31 containing pLac1	In this study
P*_*glgC*_*-LacS	*S. acidocaldarius* MR31 containing pLac2	In this study
P*_*amyA*_*-LacS	*S. acidocaldarius* MR31 containing pLac3	In this study
Plasmids		
pC	*E. coli*–*S. acidocaldarius* shuttle vector	Berkner et al., 2007
pKHmalA	His_6x_-MalA expression vector, pC derivative, containing P*_*gdhA*_* fused with *malA* gene from *S. acidocaldarius* DSM639	Choi et al., 2013
pLac1	pKHmalA derivative, containing P*_*glgP*_* fused with *lacS* from *S. solfataricus* P2	In this study
pLac2	pKHmalA derivative, containing P*_*glgC*_* fused with *lacS* from *S. solfataricus* P2	In this study
pLac3	pKHmalA derivative, containing P*_*amyA*_* fused with *lacS* from *S. solfataricus* P2	In this study
pΔ*glg*	Suicide vector for constructing *glg* operon deletion mutant, pGEM-T derivative, containing upstream, downstream of *glg* operon and *pyrE* gene from *S. solfataricus*	In this study
pΔ*glgA*	Suicide vector for constructing *glgA* deletion mutant, pGEM-T derivative, containing upstream, downstream of *glgA* gene and *pyrE* gene from *S. solfataricus*	In this study
pΔ*amyA*	Suicide vector for constructing marker-less *amyA* gene deletion mutant, pGEM-T derivative, containing upstream, downstream of *amyA* gene and *pyrE* gene from *S. solfataricus*	In this study
pC::*amyA*	Expresssion vector for constructing *amyA* complementation strain, pC derivative, containing upstream of *amyA*, *amyA* gene, and downstream of *gdhA* gene (*saci_0155*).	In this study
pΔ*glgP*	Suicide vector for constructing *glgP* deletion mutant, pGEM-T derivative, containing upstream, downstream of *glgP* gene and *pyrE* gene from *S. solfataricus*	In this study
pΔ*glgX*	Suicide vector for constructing *glgX* deletion mutant, pGEM-T derivative, containing upstream, downstream of *glgX* gene and *pyrE* gene from *S. solfataricus*	In this study
pΔ*gaa*	Suicide vector for constructing *gaa* deletion mutant, pGEM-T derivative, containing upstream, downstream of *gaa* gene and *pyrE* gene from *S. solfataricus*	In this study

### Generation of the Deletion Mutants of *Sulfolobus acidocaldarius*

Putative genes related to the glycogen metabolism of *S. acidocaldarius* were expected by BLAST search with the enzymes studied in *E. coli* and *L. acidophilus*, and the operon which consisted of the putative genes was named as *glg* operon. To confirm the involvement of putative *glg* genes in the glycogen metabolism of *S. acidocaldarius*, five chromosomal *glg* genes, namely *glgA* (*saci_1201*, GenBank No. AAY80548.1), *gaa* (*saci_1198*, GenBank No. AAY80545.1), *glgX* (*saci_1197*, GenBank No. AAY80544.1), *glgP* (*saci_0294*, GenBank No. AAY79710.1), and *amyA* (*saci_1200*, GenBank No. AAY80547.1), were disrupted by insertional deletion mutation, except the *amyA* gene. The upstream and downstream regions of the target gene were amplified by PCR and fused into one fragment with *Bam*HI restriction enzyme site by overlap PCR. The fused fragment was cloned into the suicide vector (pGEM-T) and the *pyrE* gene, amplified from the genomic DNA of *S. solfataricus* P2. This fragment was inserted between the upstream and downstream regions by restriction with *Bam*HI and ligation. The *S. acidocaldarius* MR31 was transformed with the constructed vector using electroporation. Genetic disruption by the insertion of *pyrE* gene was confirmed by PCR, and the mutants were grown in the YT medium without uracil for eliminating the plasmid fragment.

The *amyA* gene was partially deleted by the markerless deletion method as the gene is present in the upstream region of *glgA* gene, which can cause the inactivation of *glgA* gene by polar effect. To construct the Δ*amyA* mutant, markerless deletion was conducted by slightly modifying the method of Wagner et al. (2012). The middle region of *saci_1200* gene (GOI fragment) and the downstream region of *saci_1200* gene (R fragment) were amplified and fused by overlapping PCR. The fused fragment, which harbored *Bam*HI restriction site between the GOI and R fragment, was cloned into the pGEM-T vector. Then, the constructed vector was cut with *Bam*HI. The upstream region of *saci_1200* gene (L fragment) and *pyrE* gene (E fragment) of *S. solfataricus* were also amplified and fused by overlapping PCR, further resulting in the construction of E-L fragment, that has *Bam*HI restriction site on both the regions. The E–L fragment was digested with *Bam*HI and ligated into the pGEM-T vector harboring GOI-L fragment to make GOI–E–L–R sequence. From the constructed vector, the E–L–R fragment was amplified and ligated into pGEM-T vector, thereby resulting in the construction of pΔ*amyA* vector. A constructed vector was inserted into the chromosomal DNA of *S. acidocaldarius* MR31 by homologous recombination at the L or R region.

For the construction of *amyA* complementation strain, promoter and *saci_1200* gene (P-*saci_1200*) was amplified. Terminator region was amplified from the downstream of *gdhA* (*saci_0155*), and fused with P-*saci_1200* fragment, resulting in construction of P-*saci_1200*-ter fragment. The fragment was treated by *Sac*II and ligated into pC vector. Constructed vector was transformed into *amyA* deletion mutant, resulting in the construction of the *amyA* complementation strain.

### Quantification of Glycogen

*Sulfolobus acidocaldarius* MR31 and deletion mutants were grown in the YT medium supplemented with 0.2% of glucose or any other specific sugar, and 20 μg/ml of uracil was added when needed. The cell growth was measured by OD of 600 nm and growth curve was fitted to sigmoid equation with three parameters. The cells were harvested at 18, 30, 42, 60, 78, and 96 h, respectively. The samples for measuring glycogen contents were prepared by the following steps: washing with buffer A (20 mM of sodium phosphate, pH of 7.4, 450 mM of NaCl) three times, lysis by sonication, removal of the cell debris by centrifugation (2,000 × *g*, 10 min, and 4°C) and separation of soluble and membrane-bound particles by centrifugation (16,000 × *g*, 40 min, and 4°C). The membrane-bound fractions were resuspended to buffer A with the same volume of soluble fractions. For the normalization of each sample, the protein concentration of each cell before the separation of soluble and membrane-bound fractions was measured by the Bradford assay (Bradford, 1976).

The content of glycogen was quantified with minor modification as described in previous research works (Dauvillée et al., 2005; Hwang et al., 2013). In total, 0.35 units of amyloglucosidase (Sigma Aldrich, St. Louis, MO, United States) was incubated with 20 μL of samples in 100 μL of 50 mM sodium acetate buffer (pH 4.5) for 30 min at 60°C. The enzyme reaction was quenched by incubating at 100°C for 5 min, followed by centrifugation to remove proteins. Half of the reaction mixture or proper dilution portion was added to 100 μL of glucose oxidase/peroxidase reagent (Sigma Aldrich) and incubated at 37°C for 30 min. The enzyme reaction was stopped by the addition of 100 μL of 6 M H_2_SO_4_. The glucose released from glycogen by the reaction of amyloglucosidase was determined colorimetrically at 540 nm. The content of glycogen was calculated by comparing the standard of glucose equivalent that was released from glycogen from oyster (Sigma Aldrich) with known concentrations ranging from 0 to 3 μg. The calculated glycogen content was normalized by the protein concentration measured from the same volume of sample to exclude effect of the number of cells in the reaction mixture. To exclude the false-positive reaction of intracellular sugars on the glycogen content measurement, the same reaction without amyloglucosidase was conducted as control, followed by glucose oxidase/peroxidase reaction. The glucose equivalent measured from the serial reaction without amyloglucosidase was termed as pseudoglycogen and used for the normalization of glycogen content in the soluble fraction. Each value was calculated from two independent samples.

### Measurement of α-Amylase Activity

The MR31 strain and *amyA* gene deletion strain were grown in the YT medium supplemented with 0.2% glucose. The cells were harvested by centrifugation (2,000 × *g*, 30 min, and 4°C) when OD reached around 0.9, and resuspended in a buffer of 20 mM sodium phosphate (pH 7.4) with 0.5 M sodium chloride, followed by lysis through the sonication and fractionation of cell-free extract. To indirectly detect the α-amylase activity, 1 mg of soluble fraction was incubated overnight with 0.1% of glycogen in 50 mM sodium acetate buffer (pH 4.5) at 75°C. The reducing sugars released from the glycogen was detected using 3,5-dinitrosalicylic acid solution and detected colorimetrically at 575 nm. The released product from the reaction with soluble fraction of each strain was compared to the standard curve of reducing sugar calculated with glucose. The experiment was conducted with triplicates.

Branch chain-length distribution of glycogen after treatment of the cell-free extract of MR31 and Δ*amyA* mutant was examined by MALDI-TOF mass spectrometry. After treatment on glycogen, the samples were precipitated with 3 volumes of ethanol for an hour and the precipitants were collected by centrifugation (16,000 × *g*, 40 min, and 4°C) and dried. The dried samples were treated with 10 U of isoamylase (Megazyme, Chicago, United States) in 50 mM sodium acetate buffer (pH 4.0) for 72 h at 40°C. The reaction was quenched by boiling for 10 min and the samples were filtered through 0.2 μm pore. MALDI-TOF analysis was conducted by the method described by Broberg et al. (2000) with modification. Samples were mixed with the same volume of 2,5-dihydroxybenzoic acid (20 mg/ml in water/acetonitrile, 1:1) and dried on the target plate (MTP 384 target plate ground steel BC. #8280784, Bruker). Bruker Autoflex Max (Bruker, MA, United States) was utilized for the MALDI-TOF with linear positive mode and the mass spectrum was detected ranging from 400 to 3,500 m/z. Calibration was done with peptide calibrant (Bruker, MA, United States) and maltoheptaose (Sigma, St. Louis, United States). The intensity of peaks, which is responsible for the degree of polymerization (DP), were integrated and distribution of DP was calculated. As mass spectra showed regular pattern that increases molecular mass of 162, which is a molecular weight of glucose in maltooligomer, mass spectra were converted to DP using maltoheptaose as a standard.

### Promoter Assay

To determine the strength of putative promoter that lies in the upstream regions of genes related to glycogen metabolism, the cells that harbor the β-galactosidase gene fused with the putative promoter region were cultivated in the YT medium supplemented with 0.4% glucose. The cells were harvested by centrifugation at overall growth phases and resuspended in buffer A and lysed by sonication. The lysed cells were fractioned by the method as described in glycogen quantification. The β-galactosidase activity was measured by using p-nitrophenyl β-glucopyranoside (pNPG) as a substrate. The assay was conducted in 100 μL of 50 mM sodium acetate buffer (pH 5.0) containing 5 mM of pNPG and 12 μg of soluble cell-free extract. The reaction mixture was incubated at 80°C for 2 min and immediately stopped by addition of 100 μL of 1 M sodium carbonate. The absorbance of pNP released from pNPG was measured at 420 nm. One unit of the enzyme was determined as the amount of enzyme that produces 1 μmol of pNP per minute. The experiment was conducted with triplicates.

### Cell Death Rate Measurement

The cell death rates of the wild type MR31 as well as the glycogen abundant and deficient mutant strain were compared in the nutrient starvation condition. To compare the death rate, each cell was grown in 400 ml of the YT medium supplemented with 0.2% glucose at 77°C. The cells were then harvested and washed twice with the Brock’s basal medium when the OD reaches at 0.4–0.5. The washed cells were inoculated into 100 ml of fresh Brock’s basal medium (without tryptone nor sugar) with the final OD of 0.5. The cell growth was monitored every 6 or 12 h by measuring the OD. The decreased rate of OD after inoculation into the fresh medium was calculated by simple regression using the OD and time at Y- and X-axis, respectively. The rate of regression was negatively converted and termed as deathrate.

(1) $$\mathrm{Death}\mathrm{rate}=-\left(\frac{△\mathrm{OD}}{△h}\right)$$

Viable cells were counted by plating. After the serial dilution, cells were dropped on plate supplemented with 0.2% tryptone and 0.2% glucose. After the incubation of 3 days, colonies which can be detected by naked eyes were counted. A 3-(4,5-dimethylthiazol-2-yl)-2,5-diphenyltetrazolium bromide (MTT) assay was conducted to compare the relative viable cells in each time points. Method of MTT assay was modified from the MTT assay conducted in bacteria (Grela et al., 2018). A total of 3 ml of cultured cells were harvested and 1 mM MTT was incubated with the cells in 100 mM sodium acetate (pH 4.5) buffer for 20 min at 77°C. Supernatants were removed by centrifugation (16,000 × *g*, 1 min, and 4°C), and formazan was solubilized in isopropyl alcohol and measured colorimetrically at 550 nm.

Additionally, the morphology of MR31, *glg* deletion strain, and *glgX* deletion strain in stress conditions were visualized by Scanning Electron Microscopy (SEM). Samples were prepared by the method of Zhang et al. (2019) with slight modification. A total of 10 μl of cell cultures were dropped on cover glass and dried. Fixation and wash steps were conducted as described in the study of Zhang et al. but the final step using a critical point dryer was excluded. Samples were coated with Pt and imaged equipped with a Zeiss GeminiSEM 500 (Carl Zeiss, Oberkochen, Germany) at 5 kV with various magnifications.

### Statistical Analysis

The difference between two samples was confirmed by *t*-test. To compare the death rate among *S. acidocaldarius* MR31, Δ*glg*, and Δ*glgX* mutants, one-way ANOVA was used, and further the Tukey test was used for *post-hoc* analysis. For the comparison of β-galactosidase activity depending on growth phase and promoter, two-way ANOVA was conducted, and the Tukey test was introduced for *post-hoc* analysis.

## Results

### *Sulfolobus acidocaldarius* Accumulates Glycogen During Growth With Various Carbon Sources

**FIGURE 1:**
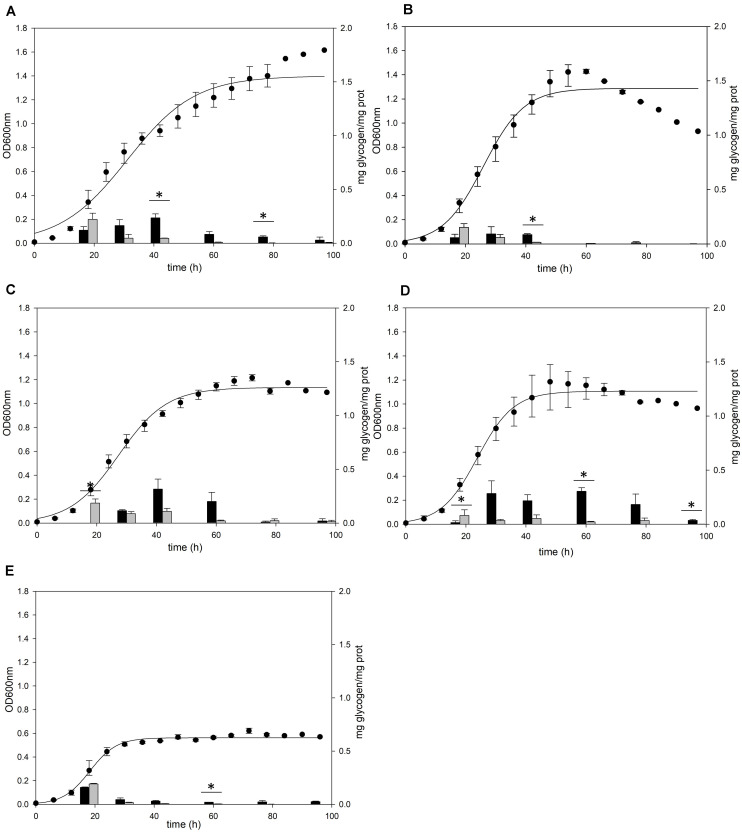
Cell growth and glycogen content of *S. acidocaldarius* MR31 grown with various sugars. Each cell was grown in the YT medium supplemented with 0.2% **(A)** glucose, **(B)** xylose, **(C)** sucrose, **(D)** dextrin, and **(E)** without sugar. *S. acidocaldarius* MR31 was inoculated in 100 ml of YT media with an initial optical density (OD) of 0.01. The cell growth was measured at an OD of 600 nm every 6 h, whereas the glycogen content of cell was calculated at 18, 30, 42, 60, 78, and 96 h. Black and gray bars present the glycogen content of soluble and membrane-bound fractions, respectively, and the content was normalized by the protein concentration. Maximum and minimum values are presented with error bars. The significant difference between glycogen content of soluble and membrane-bound fraction at each growth phase was indicated by asterisks with *p*-value < 0.05 according to *t*-test.

A previous study (König et al., 1982) detected glycogen in *S. acidocaldarius* as granular form aggregated with several proteins including GlgA. To confirm the cellular location of glycogen, *S. acidocaldarius* cells were harvested at each growth point and the cellular components in the cells were separated into soluble and membrane-bound fractions, respectively. As *S. acidocaldarius* has been known to synthesize glycogen in various carbon sources (König et al., 1982), we measured the glycogen content of *S. acidocaldarius* MR31 in the presence of different sugars. The cells were grown in the YT medium supplemented with 0.2% of glucose, xylose, sucrose, and dextrin, respectively, and the glycogen content in each cell was analyzed. As shown in Figure 1, *S. acidocaldarius* MR31 grown in different sugars accumulated glycogen in the exponential growth phase and the accumulated glycogen gradually decreased afterward. *S. acidocaldarius* MR31 accumulated 0.12 and 0.22 mg of glycogen in soluble and membrane-bound fraction per 1 mg of protein, respectively, at the early exponential phase when 0.2% glucose was provided. The glycogen content was maintained at an average of 0.27 mg of glycogen until the middle exponential growth phase and dropped to an average of 0.06 mg in the late exponential and stationary growth phases. The ratio of glycogen content in the soluble glycogen per membrane-bound glycogen (s/m) increased from 0.5 to 21.4 during the cell growth. Later, this ratio dropped to 7.0 at the stationary phase, thereby suggesting that the glycogen content in membrane-bound fraction decreased more drastically during the cell growth.

The cells grown in the YT medium supplemented with glucose (Figure 1A), sucrose (Figure 1C), and dextrin (Figure 1D) showed similar glycogen contents at an early exponential phase and a similar decline pattern. During the cell growth from the early log to middle exponential growth phase, *S. acidocaldarius* MR31 accumulated an average of 0.27 and 0.23 mg of glycogen on the supplementation of sucrose and dextrin, respectively. However, the cells grown with xylose (Figure 1B) have less glycogen content than the cells grown in the YT medium supplemented with glucose. Although *S. acidocaldarius* MR31 accumulated more than 0.2 mg of glycogen, the cells grown with 0.2% xylose accumulated an average of 0.15 mg of glycogen. *S. acidocaldarius* MR31 grown in the YT medium without sugar accumulated an average of 0.15 mg of glycogen during early and middle exponential phases (Figure 1E), indicating that *S. acidocaldarius* does not utilize xylose for the glycogen accumulation. The accumulated glycogen in the cells without sugar declined dramatically to 0.06 mg of glycogen at 30 h, suggesting that the accumulated glycogen of *S. acidocaldarius* MR31 grown without sugar was consumed rapidly compared to the cells grown with various sugars. The differences of carbon sources appeared to be the reason of less glycogen accumulation in the grown cells with xylose as compared to the cells supplemented with glucose, sucrose, and dextrin.

### *glg* Operon Plays an Essential Role in Glycogen Accumulation

**FIGURE 2:**
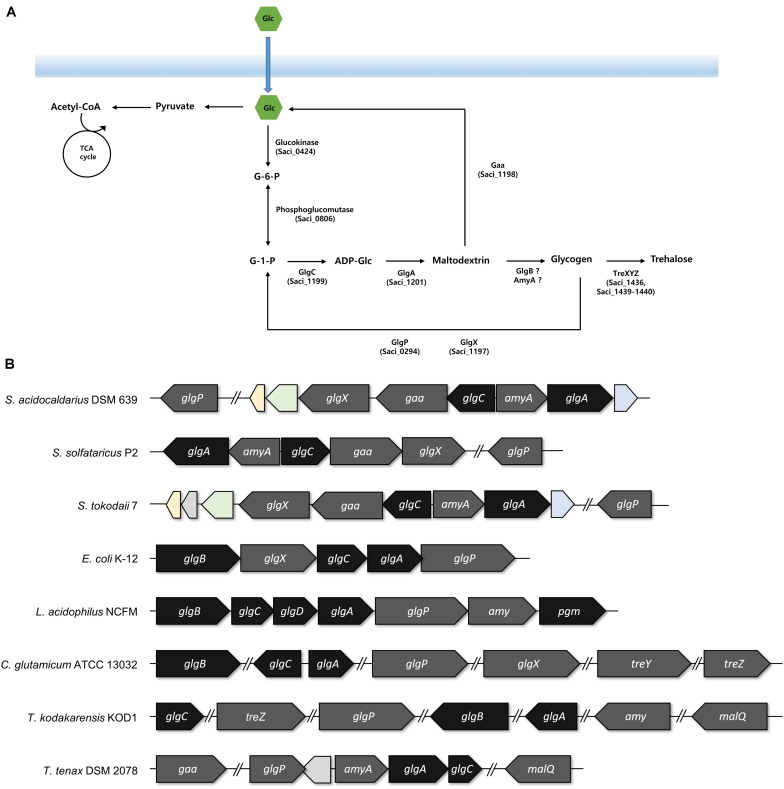
The arrangements of the genes involved in the glycogen metabolism. **(A)** Graphical scheme of glycogen metabolism in *S. acidocaldarius.* **(B)** The putative *glg* operon of *S. acidocaldarius* was compared to the arrangement of *glg* operon in *S. solfataricus*, *S. tokodaii*, *Escherichia coli*, *Lactobacillus acidophilus*, *Thermoproteus tenax*, and the genes in *Corynebacterium glutamicum* and *Thermococcus kodakarensis*. The genes responsible for the synthesis and degradation of glycogen were colored with black and gray, respectively. Homologs between *S. acidocaldarius* and *S. tokodaii* were colored; 2-iminobutanoate/2-iminopropinoate deaminase, CBS domain protein, and oxidoreductase molybdopterin binding domain containing protein was colored in yellow, green, and blue, respectively.

For identifying the gene cluster responsible for the glycogen metabolism, the *glg* operon of well-studied strains was compared with the putative *glg* operon of *S. acidocaldarius*. In *Escherichia coli*, the *glg* operon comprises five genes, namely *glgB*, *glgX*, *glgC*, *glgA*, and *glgP*. The gene cluster of *Lactobacillus acidophilus* harbors seven genes, and the *amy* gene acts as an α-amylase/glycogen debranching enzyme, thereby replacing the activity of the *glgX* gene in *E. coli* (Goh and Klaenhammer, 2013). The putative enzymes related to glycogen metabolism in *S. acidocaldarius* DSM639 are encoded by gene clusters consisting of six genes, namely *glgX* (*saci_1197*), *gaa* (*saci_1198*), *glgC* (*saci_1199*), *amyA* (*saci_1200*), *glgA* (*saci_1201*), and *glgP* (*saci_0294*), which are annotated as the putative glycogen debranching enzyme, glucoamylase, glucose-1-phosphate adenyltransferase, α-amylase, GlgA, and GlgP, respectively. Unlike the gene arrangement in *E. coli* (Serres et al., 2001) or *L. acidophilus* (Goh and Klaenhammer, 2013), the *glg* operon in *S. acidocaldarius* is divided into two operons and the putative *glgP* gene locates far from the *glg* gene clusters (Figure 2). The gene arrangement of *S. acidocaldarius* was same as the arrangement of putative *glg* operon of *S. solfataricus* (She et al., 2001) and *S. tokodaii* (Kawarabayasi et al., 2001), suggesting that the *glg* operon is conserved in related species. *S. acidocaldarius* and *S. tokodaii* also shares homologs which were predicted as 2-iminobutanoate/2-iminopropanoate deaminase (*saci_1195, STK_08110*), CBS domain protein (*saci_1196, STK_08130*), and a protein which contains oxidoreductase molybdopterin binding domain (*saci_1202, STK_08190*). Interestingly, *Thermoproteus tenax*, which is a member of Crenarchaeota, harbors *amyA* gene and lacks *glgB* gene like *Sulfolobus*, although the gene arrangement was different (Siebers et al., 2004). Unlike *T. tenax*, *Thermococcus kodakarensis*, which is a member of Euryarchaeota, was shown to have the *glgB* gene searched *via* BLAST using GlgB of *E. coli*.

The result of the reverse transcription PCR showed that the *glgC*, the *gaa*, and the *glgX* genes were co-transcribed as an operon, and the *amyA* and the *glgA* genes were also synthesized in a single mRNA transcript (see Supplementary Figure 1). To identify the conserved region among the promoters of *glgP*, *glgC–gaa–glgX*, and *amyA–glgA*, WebLogo was used with 50 bp upstream of *saci_0294* (*glgP*), *saci_1199* (*glgC*), and *saci_1200* (*amyA*) (Crooks et al., 2004). The putative promoter of *glgX*, *amyA*, and *glgP* has conserved A/T rich sequences on the -31 to -26 bp upstream of the start codon (see Supplementary Figure 2A). Intergenic region of *gaa–glgX*, which could be a promoter region for *glgX* also has the conserved A/T rich sequences (see Supplementary Figure 2B). The *glgX* gene, which lies 172 bp upstream region of the *gaa* gene, is shown to also have its own promoter, further making it possible to be transcribed alone. The scattered location of the genes related to the glycogen metabolism also existed in *C. glutamicum* (Ikeda and Nakagawa, 2003). Unfortunately, the putative GlgB, which is encoded by the *glgB* gene and forms α-1,6-linked branches of glycogen, was not found in *S. acidocaldarius* by the BLAST search with GlgB of *E. coli* or *L. acidophilus*.

**FIGURE 3:**
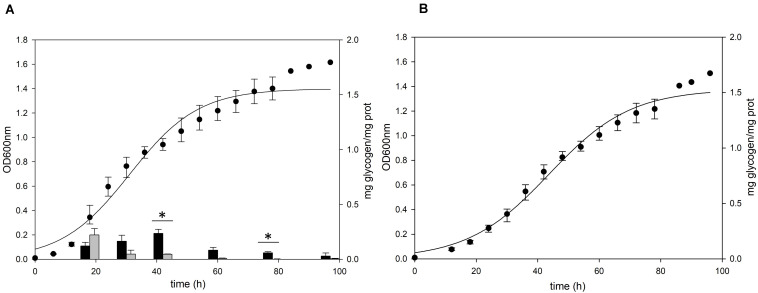
Growth and glycogen content of *S. acidocaldarius* MR31 and Δ*glg* mutant. **(A)** MR31 and **(B)** Δ*glg* strains were grown in the YT media supplemented with 0.2% glucose. The cells were inoculated to media with the initial OD of 0.01. Black and gray bars represent the glycogen content of soluble and membrane-bound fractions, respectively, and the content was normalized by the protein concentration. Error bars indicate maximum and minimum values. The significant difference between glycogen content of soluble and membrane-bound fraction at specific growth phase was indicated by asterisks with *p*-value < 0.05 according to *t*-test.

To confirm the role of the putative *glg* operon in the glycogen metabolism of *S. acidocaldarius*, the glycogen content of *glg* operon deletion strain was compared to that of the MR31 strain. For constructing the *glg* deletion mutant, the gene cluster reaching from *glgX* to *glgA* was removed by insertional deletion with the *pyrE* gene (see Supplementary Figure 3). While *glg* deletion mutant showed a slower increase of absorbance at 600 nm in the early log phase, the maximum OD was similar between the two strains (Figure 3). Despite few differences in the cell growth, no glycogen was detected in both of membrane-bound and intracellular fractions of the *glg* mutant, thereby suggesting that the putative *glg* operon plays a crucial role in the glycogen metabolism of *S. acidocaldarius.*

### Deletion of *glgA* or *amyA* Genes Does Not Result in Glycogen Accumulation in *Sulfolobus acidocaldarius*

**FIGURE 4:**
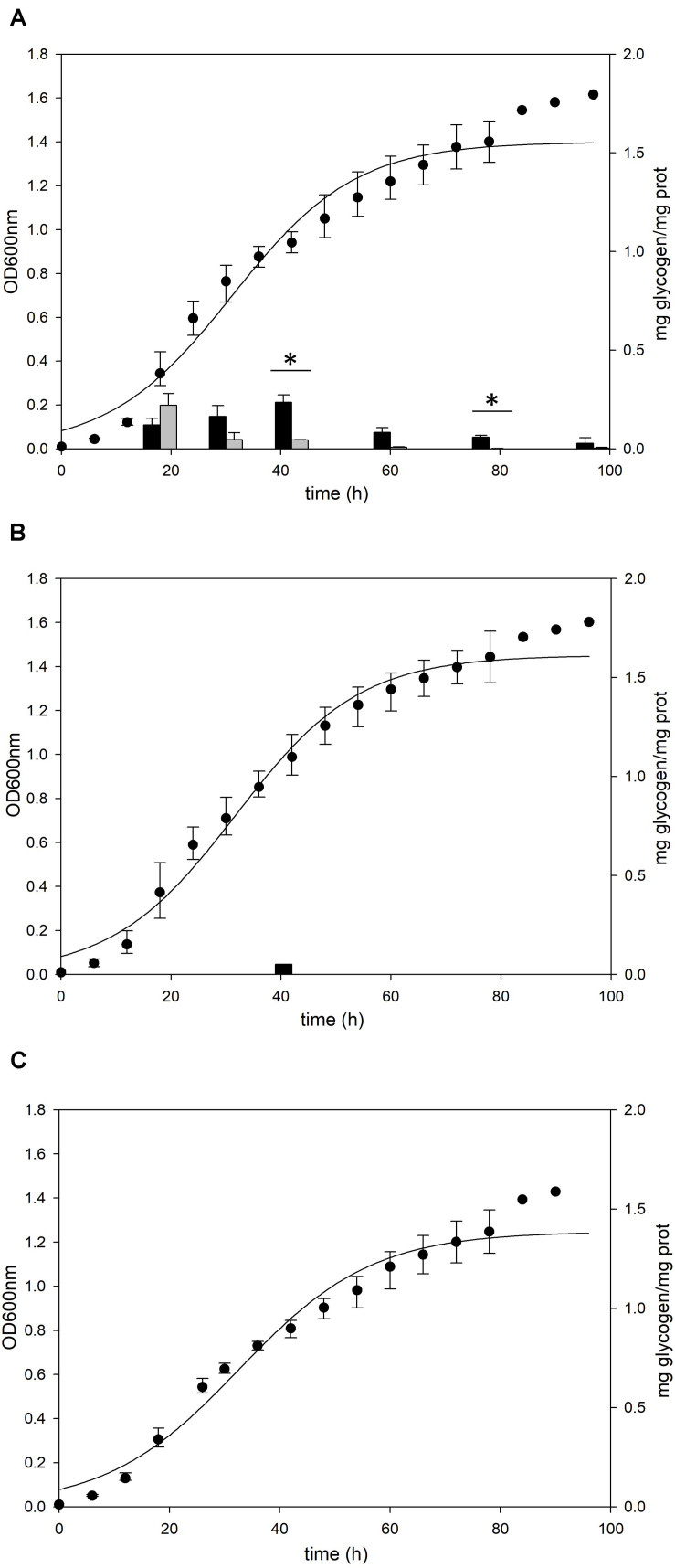
Growth and glycogen content of MR31, Δ*glgA*, and Δ*amyA* mutants. To elucidate the roles of GlgA and AmyA in glycogen metabolism, the cell growth and glycogen content of **(A)** MR31, **(B)** Δ*glgA*, and **(C)**Δ*amyA* mutant were compared. Each strain was inoculated to the YT media supplemented with 0.2% glucose with the initial OD of 0.01 and the growth and glycogen content were detected. OD was detected every 6 h, and the cells were harvested at 18, 30, 42, 60, 78, and 96 h for the quantification of cytosolic (black) and membrane-bound (gray) glycogen. The significant difference between glycogen content of each fraction at the same time point was represented by asterisks with *p*-value < 0.05.

To identify the functional roles of GlgA and AmyA in glycogen metabolism, each gene was disrupted by insertional deletion and markerless mutation, respectively. Each deletion mutant was grown in the YT medium supplemented with 0.2% glucose and the content of intracellular glycogen was measured in various growth phases. Unlike MR31 strain (Figure 4A), both strains did not show the accumulation of intracellular glycogen *via* cell growth (Figures 4B,C). As expected, the *glgA* deletion mutant showed the lack of accumulated glycogen, as *glgA* encodes glycogen synthase, which acts as a key enzyme in the formation of α-1,4-glycosidic chain.

Unlike the amino acid sequence-based expectation that the *amyA* gene encodes α-amylase, which is predicted to be involved in the degradation of glycogen, the mutant with the *amyA* gene deletion failed to accumulate the intracellular glycogen (Figure 4C). When the vector that can express *amyA* gene was transformed to *amyA* deletion mutant, the complement cells accumulated glycogen again (see Supplementary Figure 4), suggesting that AmyA plays an essential role in glycogen synthesis.

**FIGURE 5:**
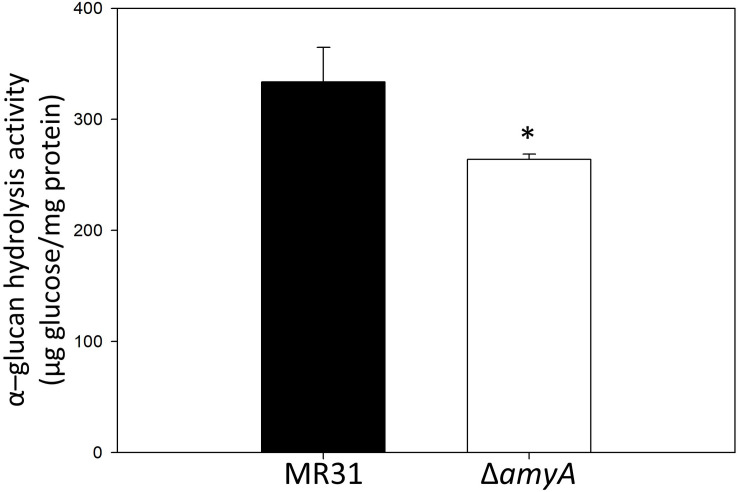
Comparison of the glycogen hydrolase activity of MR31 and Δ*amyA* mutant. *S. acidocaldarius* MR31 and Δ*amyA* mutant were grown in the YT media supplemented with 0.2% glucose, and the cells were harvested, and lysed. Thereafter, the soluble protein was fractioned. The hydrolysis activity of soluble fraction toward glycogen was measured using glycogen as a substrate. Reactions were conducted at 75°C. The experiment was conducted with triplicates, and asterisks represents *p*-value < 0.05.

To purify and characterize the AmyA activity, various expression systems were used, but all the expression systems used for the expression of *amyA* gene in *S. acidocaldarius* or *E. coli* failed to do the same. To indirectly identify the enzyme activity of AmyA, the hydrolysis abilities of the glycogen by using total cell-free extract in the MR31 strain and *amyA* deletion strain were compared. Figure 5 shows that the cell-free soluble fraction of MR31 had a higher hydrolytic activity as compared to the Δ*amyA* mutant. This result indirectly suggests that AmyA has the capacity to hydrolyze glycogen.

The role of AmyA was further examined by treatment of glycogen with MR31 and Δ*amyA* mutant. Branch chain-length distribution of glycogen after treatment of total cell-free extract of MR31 strain and Δ*amyA* mutant was detected by MALDI-TOF. As shown in Supplementary Figure 5, chain-length distribution of glycogen was changed after treatment of both extracts. When glycogen was treated with cell-free extract of MR31, the percentage of maltopentaose (DP5) was increased compared to the untreated glycogen and the distribution of branches with DP over 10 was decreased. The branches which comprise more than DP16 were disappeared. However, when the glycogen was treated with the cell-free extract of Δ*amyA* mutant, chain distribution with DP over 10 was complemented and branches were detected until DP18, suggesting that AmyA possesses the hydrolytic activity toward over DP10. The role of AmyA in the glycogen metabolism will be discussed below.

### Gaa and GlgX Play a Major Role in the Degradation of Glycogen

**FIGURE 6:**
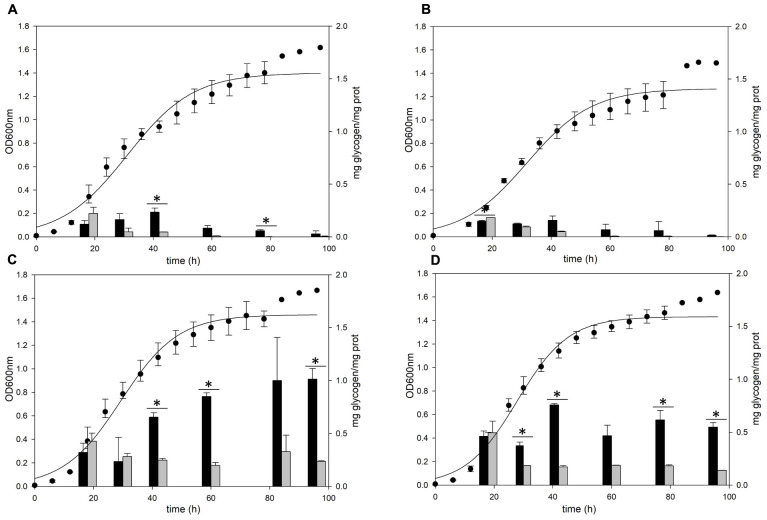
Growth and glycogen content of MR31, Δ*glgP*, Δ*glgX*, and Δ*gaa* mutants. To elucidate the roles of GlgP, GlgX, and Gaa in glycogen metabolism, the cell growth and glycogen content of **(A)** *S. acidocaldarius* MR31, **(B)** Δ*glgP*, **(C)** *glgX*, and **(D)** Δ*gaa* mutant were compared. The cells were grown in the YT media supplemented with 0.2% glucose. The cell growth and glycogen content of soluble and membrane-bound fractions were represented as black and gray bars, respectively. The significant difference between glycogen content of soluble and membrane-bound fraction at each growth phase was indicated by asterisks with *p*-value < 0.05.

The *gaa, glgX*, and *glgP* genes were inactivated by insertional mutation to identify the functions in glycogen metabolism. The gene deletion strains were grown in the YT medium supplemented with 0.2% glucose and the glycogen contents in each growth phases were measured. The pattern of glycogen content in the *glgP* deletion mutant was similar to the glycogen content of MR31 strain (Figures 6A,B), hence indicating that the deletion of GlgP, predicted as a glycogen phosphorylase, results in a modest change in the glycogen content of *S. acidocaldarius*.

As compared to the MR31 strain, the amount of glycogen in *glgX* and *gaa* deletion mutants was significantly higher in all phases. The total content of soluble and membrane-bound glycogen in the *glgX* deletion mutant was approximately 0.7 mg glycogen per 1 mg protein in the early exponential phase and further increased during the cell growth. This strain finally accumulated approximately 1.2 mg glycogen per 1 mg protein in stationary phase (Figure 6C). Similar to the *glgX* deletion mutant, glycogen gradually accumulated in the *gaa* deletion mutant and reached approximately 1.0 mg glycogen per 1 mg protein in the stationary phase (Figure 6D). In both *glgX* and *gaa* deletion mutants, the intracellular glycogen was much higher than that of the MR31 strain, especially in the stationary phase. In detail, *glgX* and *gaa* deletion mutant accumulated 2.6- and 2.9-times higher glycogen content during the exponential growth phase and 38.5- and 21.2-times higher glycogen content in the stationary phase, respectively. This result suggests that GlgX and Gaa, which are predicted as glycogen-debranching enzyme and glucoamylase, respectively, play a major role in the degradation of glycogen in *S. acidocaldarius*.

### Differences in the Promoter Strength of *glg* Operon at Each Growth Phase Reflect the Change in the Glycogen Content of *S. acidocaldarius* During the Cell Growth

**FIGURE 7:**
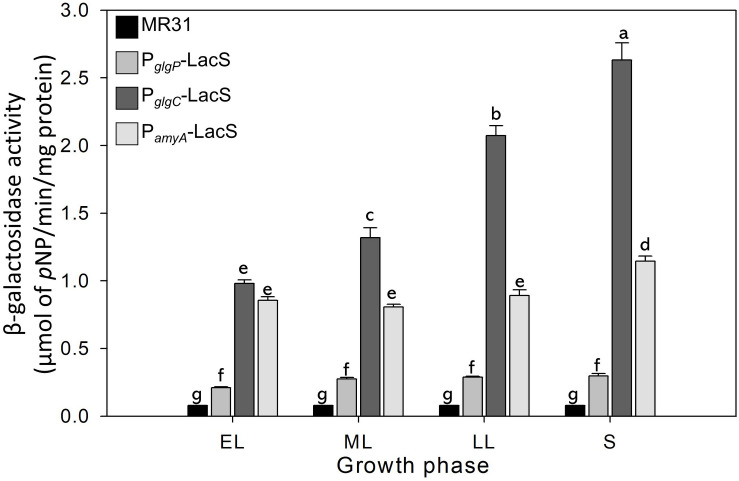
The promoter strength of *glgP*, *gaa*, and *amyA* of *S. acidocaldarius* according to the growth phase. MR31 strain and mutants, which obtain the *lacS* gene of *S. solfataricus* that is fused with each promoter by transformation, was grown in the YT media supplemented with 0.4% glucose. After the harvest, the cells were lysed, and soluble fractions were used to detect the β–galactosidase activity. The enzyme activity was calculated by the detection of released pNP. The strength of promoter was measured in each growth phase. EL, early log phase; ML, mid log phase; LL, late log phase; S, stationary phase. Error bars represent standard deviation calculated from triplicate experiments. The significant difference between samples was indicated by superscript letters with *p*-value < 0.05 according to the Tukey test.

The glycogen content of *S. acidocaldarius* was increased during the early exponential phase and the accumulated glycogen is decreased gradually during the late exponential and stationary growth phases. The transcriptional analysis was conducted to investigate the changes of glycogen content during the growth. Three putative promoters located in the upstream regions of *amyA–glgA*, *glgC–gaa–glgX* operon, and *glgP* gene were fused with the β-galactosidase (LacS) gene amplified from the chromosomal DNA of *S. solfataricus* P2. All *S. acidocaldarius* strains that contains the *lacS* gene, fused with a putative promoter region, showed a higher β-galactosidase activity as compared with MR31 strain (Figure 7). The *S. acidocaldarius* strain, which harbors the *lacS* gene fused with the putative promoter of *glgP* or *amyA*, showed a constant β-galactosidase activity regardless of the growth phase. In contrast, the enzyme activity of the strain harboring the *lacS* gene fused with the putative promoter of *glgC* gradually increased during cell growth, thereby suggesting that the expressions of *amyA* and *glgA* are constant in the overall cell growth but the expressions of *glgC, gaa*, and *glgX* increase along the cell growth. The result of promoter assay can be account for the gradual decrease of glycogen content during the cell growth. The gradual increase of GlgX and Gaa, which play an important role in the glycogen degradation, is supposed to affect the glycogen content, further resulting in a gradual decrease.

### Glycogen in *S. acidocaldarius* Is Involved in the Maintenance in Harsh Conditions

**FIGURE 8:**
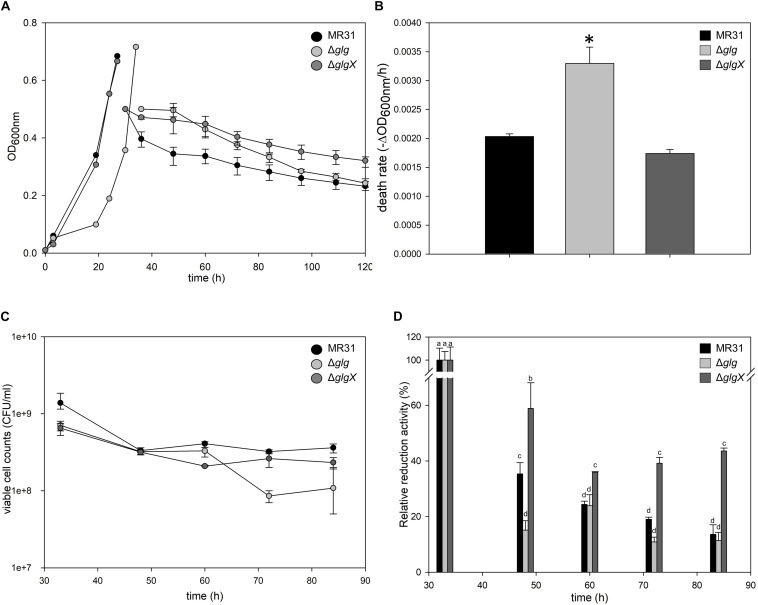
Comparison of cell death rate. **(A)** OD of MR31, Δ*glgX*, and Δ*glg* was detected. When the OD surpassed 0.6, the cells were harvested, washed, and transferred into fresh media with the initial OD of 0.5. The decreased OD rate of each cells were represented as death rate **(B)**. One-way ANOVA and Tukey test were used for the statistical analysis, and asterisks represents *p*-value < 0.05 according to the Tukey test. **(C)** Quantification of viable cells by plating. Liquid cultures of each strain were serial diluted with Brock’s basal media and dropped on YT plate. After the incubation of 3 days, colonies were counted. **(D)** Relative quantification of viable cells by MTT assay. Initial OD at 550 nm of each strain was calculated as 100%. The significant difference between samples was indicated by superscript letters with *p*-value < 0.05 according to the Tukey test.

To investigate the relationship between the intracellular glycogen and cell properties, the death rate of wild type and glycogen deficient mutants were compared in the Brock’s basal medium. The growth of *S. acidocaldarius* Δ*glg* mutant, which cannot accumulate the intracellular glycogen, was slower than the growth of wild type MR31 and *glgX* mutant strains in the early exponential growth phase (Figure 8A). The doubling times of MR31, Δ*glg*, and Δ*glgX* in an early log phase were determined as 3.72, 5.82, and 3.87 h, respectively. The mutant and wild type cells were harvested when the OD surpassed 0.5, where the glycogen content of both fractions was maintained, and each strain was inoculated into 100 ml of Brock’s basal media after washing with fresh Brock’s basal media at the initial OD of 0.5. The OD of each cell was detected every 6- or 12-h interval. The OD of Δ*Glg* mutant was decreased more rapidly than that of the MR31 strain. The death rate of each strain was calculated by using simple regression in the section, which showed a linear decrease (Figure 8B). The result indicates that the absence of glycogen affects the growth rate of *S. acidocaldarius* at an early exponential phase and the death rate when the cells were placed in nutrient deficient conditions.

To count viable cells, plating and MTT assay were conducted. CFU of MR31, Δ*glg*, and Δ*glgX* right after the inoculation into Brock’s basal media was an average of 11.5 × 10^8^, 7.0 × 10^8^, and 8.0 × 10^8^ per 1 ml of culture, respectively. After 24 h of the incubation in Brock’s basal media, CFU of MR31, Δ*glg*, and Δ*glgX* decreased to 3.6 × 10^8^, 1.0 × 10^8^, and 2.3 × 10^8^, respectively (Figure 8C). All the strain showed a reduced CFU, especially Δ*glg* showed the greatest reduction. To quantify the viable cells indirectly, MTT activity of 3 ml cultures were compared (Figure 8D). Reductase activity of each cell after inoculation into Brock’s basal media was compared to the activity before inoculation. MR31 and Δ*glg* showed similar relative activity at 24 h after the inoculation, but reductase activity of Δ*glg* decreased faster. Cell morphology before and after the inoculation into Brock’s basal media was visualized by SEM (see Supplementary Figure 6A). While MR31 and Δ*glgX* showed lysed morphology at 48 h after the inoculation, Δ*glg* already showed lysed morphology at 24 h after the inoculation, suggesting that Δ*glg* is more sensitive to nutrient starvation. In addition, Δ*glg* seemed to be more sensitive to osmotic stress as well (see Supplementary Figure 6B). From this result, we can speculate that glycogen, which is absent in the Δ*glg* mutant but present in MR31 and Δ*glgX* mutant, is involved in the maintenance of cell growth when starvation condition or stress is provided.

## Discussion

A previous study on *S. acidocaldarius* demonstrated that this hyperthermophilic archaeon accumulates intracellular glycogen in a granular form (König et al., 1982), but previous studies focused on the characterization of the enzymes which are supposed to be involved in glycogen metabolism. GlgA, which was initially identified as polyphosphate kinase with transglycosylation activity, was expressed in *E. coli* and identified as glycogen synthase (Skorko et al., 1989; Cardona et al., 2001). Gaa of *S. solfataricus* and *S. tokodaii* was characterized to be able to hydrolyze both α-1,4- and α-1,6-glycosidic linkages (Kim et al., 2004; Njoroge et al., 2005). Recombinant form of *S. solfataricus* TreX was shown to possess α-1,4-transferase activity as well as α-1,6-glucosidase activity, thereby suggested to play a role in the degradation of glycogen and synthesis of trehalose, and these properties are also detected from the TreX of *S. shibatae* (Park et al., 2007; Van et al., 2007; Woo et al., 2008). Although a couple of enzymes related to glycogen biosynthesis and degradation have been characterized in *Sulfolobus* species, the detailed studies on the glycogen metabolism have not been conducted yet. Using glucoamylase and glucose oxidase, we indirectly measured the glycogen contents in *S. acidocaldarius*. In this study, the gene clusters related to glycogen metabolism were uncovered by genetic approaches and the role of each enzyme was identified using gene deletion mutants.

We compared the glycogen content in the presence of various sugars. For sufficient cell growth, the Brock’s basal medium was supplemented with tryptone and yeast extract, which can be used for the accumulation of glycogen in cells (König et al., 1982). The glycogen was still detected in *S. acidocaldarius* MR31 in the absence of additional carbon source (Figure 1E), and this glycogen seemed to be accumulated using components of the YT medium because the addition of tryptone and yeast extract can provide sugars to *S. acidocaldarius* MR31. The glycogen content of *S. acidocaldarius* MR31 was increased when various carbon sources were provided, but the overall content was similar when glucose, dextrin, and sucrose was given as carbon sources. As *S. acidocaldarius* cannot utilize fructose as a carbon source (Chen et al., 2005), the accumulated glycogen content of *S. acidocaldarius* grown with sucrose seemed to be only affected by glucose.

*Sulfolobus acidocaldarius* can use glucose through semi-phosphorylative ED (sp ED) and non-phosphorylative ED (np ED) pathways (Quehenberger et al., 2017). In sp. ED pathway, glyceraldehyde-3-phosphate (GAP) is produced, and GAP can be used to synthesize glycogen. Pyruvate, which is released from the reaction that converts 2-keto-3-deoxy-6-phosphate-D-gluconate (KDPG) into GAP, can also convert into GAP through gluconeogenesis, and further utilized to synthesize glycogen. In the np ED pathway, two molecules of pyruvate are synthesized from one molecule of glucose, and the pyruvate can be further utilized to synthesize glycogen. When glucose is provided, *S. acidocaldarius* accumulates glycogen regardless of the pathway they use.

The glycogen content of *S. acidocaldarius* grown without sugar was similar to those of cells grown in YT medium supplemented with xylose, indicating that providing the xylose does not affect the glycogen content of *S. acidocaldarius*. *Sulfolobus* harbors two different pathways for the utilization of D-xylose and L-arabinose, Dahms pathway and Weimberg pathway (Quehenberger et al., 2017). While pyruvate is released by the reaction of KD(P)G aldolase that involves in Dahms pathway, Weimberg pathway does not form pyruvate as an intermediate. *S. acidocaldarius* DSM 639 appears to utilize both pathways equally based on the results of enzyme properties and *in vivo* metabolite labeling studies (Brouns et al., 2006; Nunn et al., 2010). However, MW001 strain, which is uracil auxotrophic derivatives of *S. acidocaldarius* DSM 639, appears to use Weimberg pathway as the transcriptional level of the genes related to Weimberg pathway, were significantly increased compared to those of the Dahms pathway in the presence of D-xylose (Wagner et al., 2018). It has been explained that the difference in pentose-utilizing pathway between DSM 639 and MW001 depends on the ability to utilize xylose as a sole carbon source (Wagner et al., 2018). As *S. acidocaldarius* MR31 was unable to grow when xylose was provided as a sole carbon source, we speculate that the lower glycogen content in MR31 strain grown with xylose is related to the preference of the Weimberg pathway which was observed in *S. acidocaldarius* MW001, resulting in the lack of pyruvate that can be further utilized into glycogen synthesis.

The synthesis of glycogen is proceeded by the reactions of GlgC, GlgA, and GlgB. The genes that encode GlgC and GlgA were identified, but the encoding gene of GlgB, which is responsible for the branching enzyme, is still unknown. A previous study of *S. solfataricus* showed that TreX, which is known as the glycogen debranching enzyme, exhibited an additional activity that transfers maltosyl residue and forms α-1,6-glycosidc linkage (Park et al., 2007). If TreX in *S. acidocaldarius* has same enzymatic properties as the enzyme of *S. solfataricus*, then this protein can substitute the activity of GlgB. Hence, it is crucial to investigate the relationship of TreX in glycogen metabolism.

Various enzymes are responsible for the degradation of glycogen. The *glg* operon of *S. acidocaldarius* harbors four putative enzymes, namely GlgP, GlgX, Gaa, and AmyA, that are related to glycogen degradation. Although the deletion of *glgP* exerted a minor effect on the glycogen content, the deletion of *glgX* and *gaa* showed a dramatic increase of glycogen. Two distinctive genes, namely, *gaa* and *amyA*, that encode α-glucan hydrolase, are located within the *glg* operon, but the deletion of each gene showed a different result. Gaa, which possess a glucoamylase activity (data not shown), cleaves α-1,4-glycosidic bonds from the non-reducing end of glycogen and releases glucose. The result of glycogen content in *gaa* deletion mutant matches with the predicted enzyme activity that plays an active role in glycogen degradation. Unlike the result of *gaa* deletion mutant, the glycogen content in *amyA* deletion mutant was opposite to the expected result based on the result of glycogen hydrolysis assay. Although *amyA* deletion mutant showed a reduced hydrolysis activity toward glycogen as compared to the wild type, no accumulated glycogen was detected in the *amyA* deletion mutant. AmyA appeared to play a crucial role in the synthesis of glycogen in *S. acidocaldarius*; hence, there is an urgent need to investigate the relationship between the glycogen content and AmyA. In *L. acidophilus*, the enzyme encoded by *amy* gene possessed both α-amylase and glycogen debranching activity (Goh and Klaenhammer, 2013). A previous study showed that GlgX in *E. coli* is active not only in the degradation but also in the synthesis of glycogen, and shapes the structure of glycogen by reducing the ratio of short chains (Dauvillée et al., 2005). We can speculate that AmyA, which is a putative α-amylase, has debranching activity and therefore plays an important role in trimming or rearranging the intermediates during the synthesis of glycogen in *S. acidocaldarius*.

The result of promoter assay shows that the gene cluster was divided into two distinguishable operons, and the genes related to the synthesis and degradation of glycogen were transcribed parallelly. The transcription of gene clusters, measured by a promoter assay, showed that the transcriptional level of *glgC-gaa-glgX* genes gradually increased during the cell growth, whereas the transcription of *glgP* and *amyA-glgA* genes remained constant throughout the entire growth phase. This result implies that the gradual decrease of glycogen after the exponential growth phase in *S. acidocaldarius* occurs due to the increase of the enzymes involved in glycogen degradation.

Glycogen metabolism is controlled by complex regulatory systems. The expression of *glgC*-*glgA*-*glgP* of *E. coli* can be activated by cAMP and CRP, and ppGpp can also activate the expression of the gene cluster (Preiss, 1984; Preiss and Romeo, 1990). Additionally, the glycogen metabolism in *E. coli* is positively controlled by PhoP–PhoQ, which is a two component regulatory system that monitors external Mg^2+^ concentration, in the limited Mg^2+^ condition (Montero et al., 2009). CsrA inhibit the glycogen synthesis in *E. coli* by binding to the *glgC* Shine-Dalgarno sequence and the *glgCAP* leader transcript (Baker et al., 2002). However, as homolog of Csr is lack in Archaea (Romeo, 1998), the regulation of glycogen metabolism *via* Csr is irrelevant to *S. acidocaldarius*. In *T. kodakarensis*, transcription of key enzymes for glycogen biosynthesis such as GlgB was increased by the deletion of *tgr*, which encodes Thermococcales glycolytic regulator, suggesting that the glycogen synthetic pathway is repressed by Tgr in the gluconeogenic conditions (Kanai et al., 2007). However, Tgr homologs were not detected in *S. acidocaldarius* by BLAST search. Since the glycogen synthesis and degradation genes are co-transcribed in our study, it is likely that the post-transcriptional regulation is important for the changes in the glycogen level during cell growth. The regulation of glycogen metabolism in Archaea has not been studied yet and still needs to be uncovered. It will be interesting to investigate the regulatory mechanism of glycogen metabolism in *S. acidocaldarius*.

The glycogen in *S. acidocaldarius* is predicted to be utilized to produce trehalose by the reaction of TreY and TreZ, which synthesize trehalose from maltodextrin (Maruta et al., 1996; De Smet et al., 2000). The glycogen in *S. acidocaldarius* can be turned into maltodextrin by the reaction of TreX, which has glycogen debranching activity (Maruta et al., 1996) and 4-α-glucanotransferase activity similar to TreX in *S. solfataricus* (Park et al., 2007). In *treXYZ* deletion mutant, which cannot synthesize trehalose using glycogen as a substrate, the amount of accumulated glycogen was slightly higher than the amount of glycogen in wild type, but comparably lower than the glycogen content in *glgX* and *gaa* deletion mutant (data not shown). This observation suggests that the role of glycogen in *S. acidocaldarius* is not just limited to being the substrate for trehalose.

When heterotrophic organisms are transferred into nutrient depleted conditions, the cells find it hard to sustain their growth unless they can utilize the stored energy and carbon sources. We hypothesized that glycogen present in *S. acidocaldarius* plays an active role in carbon and energy storage for the survival, as the mutant that cannot reserve glycogen exhibited a higher death rate than *S. acidocaldarius* MR31 in the comparison of death rates. In a previous study, the genes related to the synthesis and the degradation of glycogen decreased and increased, respectively, as the nutrient depletion condition was provided. The deficiency of nutrient increased the transcription of the genes encoding enzymes that are related to the conversion of glycogen to glucose-1-phosphate and glucose-1-phosphate into glucose-6-phosphate. Consequently, the degradation of glycogen can quickly form glucose intermediates when the environmental nutrients are limited (Bischof et al., 2019). When *E. coli* MRE 162 was grown in nitrogen-limited or carbon-limited conditions and further transferred to saline phosphate buffer without magnesium at 37°C, the cells harboring the highest amount of glycogen showed the highest survival rate (Strange, 1968). In *Vibrio cholerae*, mobilization of internal glycogen gives the survival advantages in nutrient-poor environments and pond water, one of the ecological niche of *V. cholerae* (Bourassa and Camilli, 2009). In *Salmonella enterica* Typhimurium, the *glgC* mutant survived less in saline, feces, and water than other strains with complete *glgC*, suggesting that glycogen plays an important role in survival (McMeechan et al., 2005).

The growth rate of *glg* deletion mutant in our study was also slower than that of the *S. acidocaldarius* MR31, implying that the intracellular glycogen may play a supportive role in the cell growth and maintenance. The study of *E. coli* figured out that *glgP* deletion mutant, which cannot utilize glycogen, exhibited longer lag times than the wild type when the cells meet at sudden environmental changes (Sekar et al., 2020). Dynamic study of cellular metabolism in *E. coli* suggests that intracellular glycogen is the only sugar donor in the lag phase before the cell growth (Yamamotoya et al., 2012). Based on the results of glycogen content and the promoter analysis as well as growth and death rate evaluation of the *glg* mutants, the glycogen metabolism appears to be involved in growth maintenance and effective carbon cycling in *S. acidocaldarius*.

## Data Availability Statement

The raw data supporting the conclusions of this article will be made available by the authors, without undue reservation.

## Author Contributions

AL designed and performed the experiments except mutant construction, analyzed the data, and drafted the manuscript. EB contributed to the idea development and constructed mutants for the promoter assay. JP contributed to data analysis and constructed the gene deletion mutants. K-HC conceived the study and contributed to the experimental design. JC conceived the study and revised the manuscript. All authors discussed the result and contributed to manuscript.

## Conflict of Interest

The authors declare that the research was conducted in the absence of any commercial or financial relationships that could be construed as a potential conflict of interest.
