# Supplementary figures and images for: Identification of the Genes Related to the Glycogen Metabolism in Hyperthermophilic Archaeon, Sulfolobus acidocaldarius
Source: Front Microbiol. 2021 May 13;12:661053. doi: 10.3389/fmicb.2021.661053 (PMC8158581; doi:10.3389/fmicb.2021.661053)

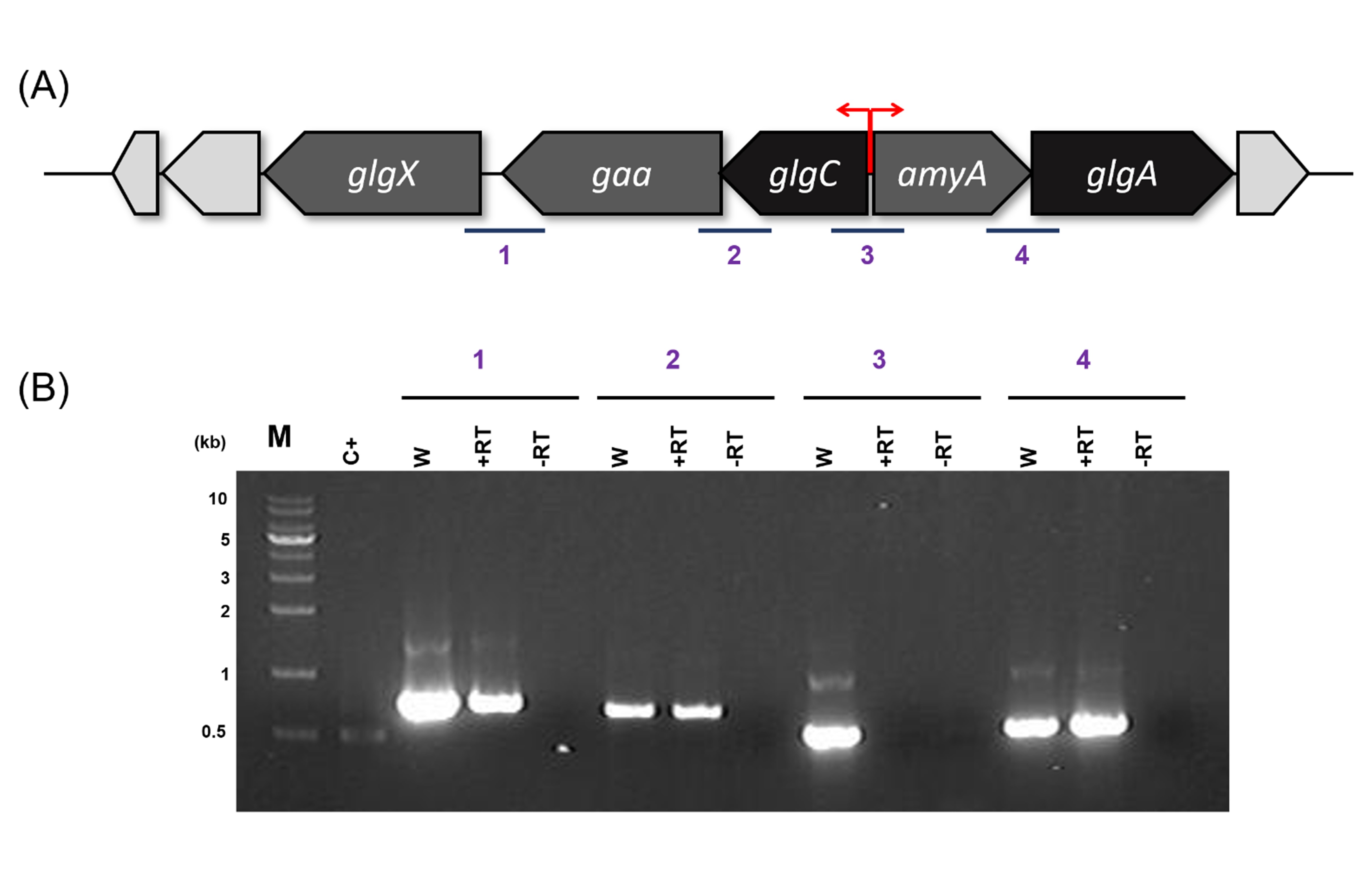

Supplement: Supplementary file 1 [file Image_1.JPEG]

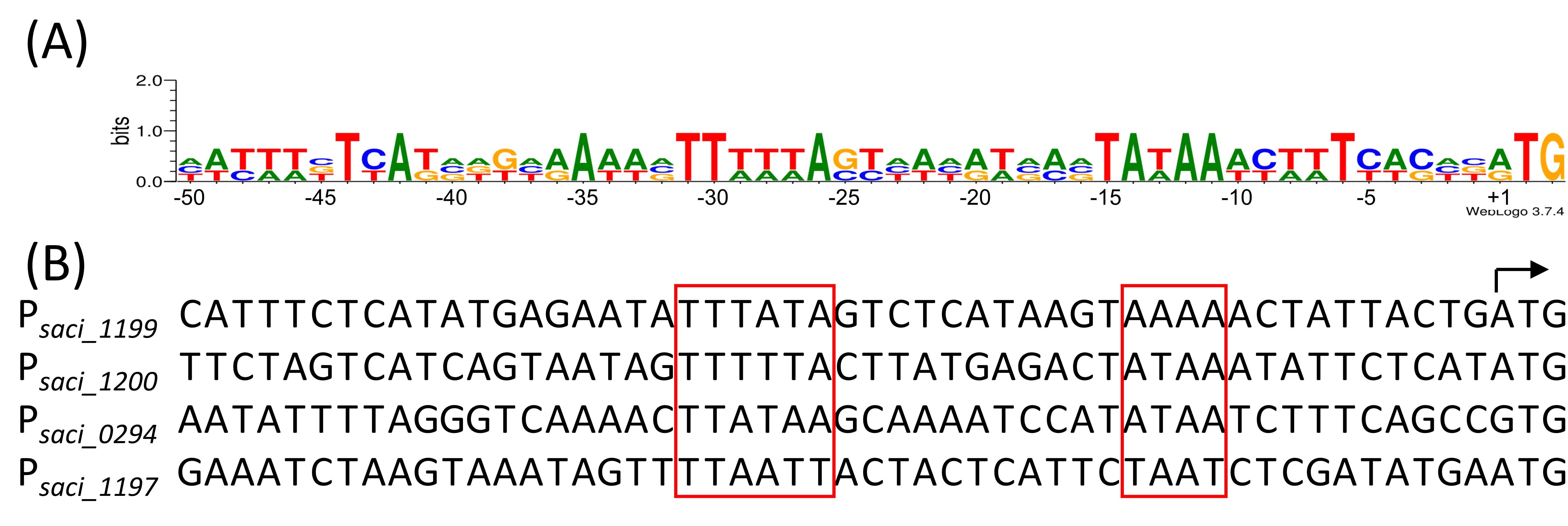

Supplement: Supplementary file 2 [file Image_2.JPEG]

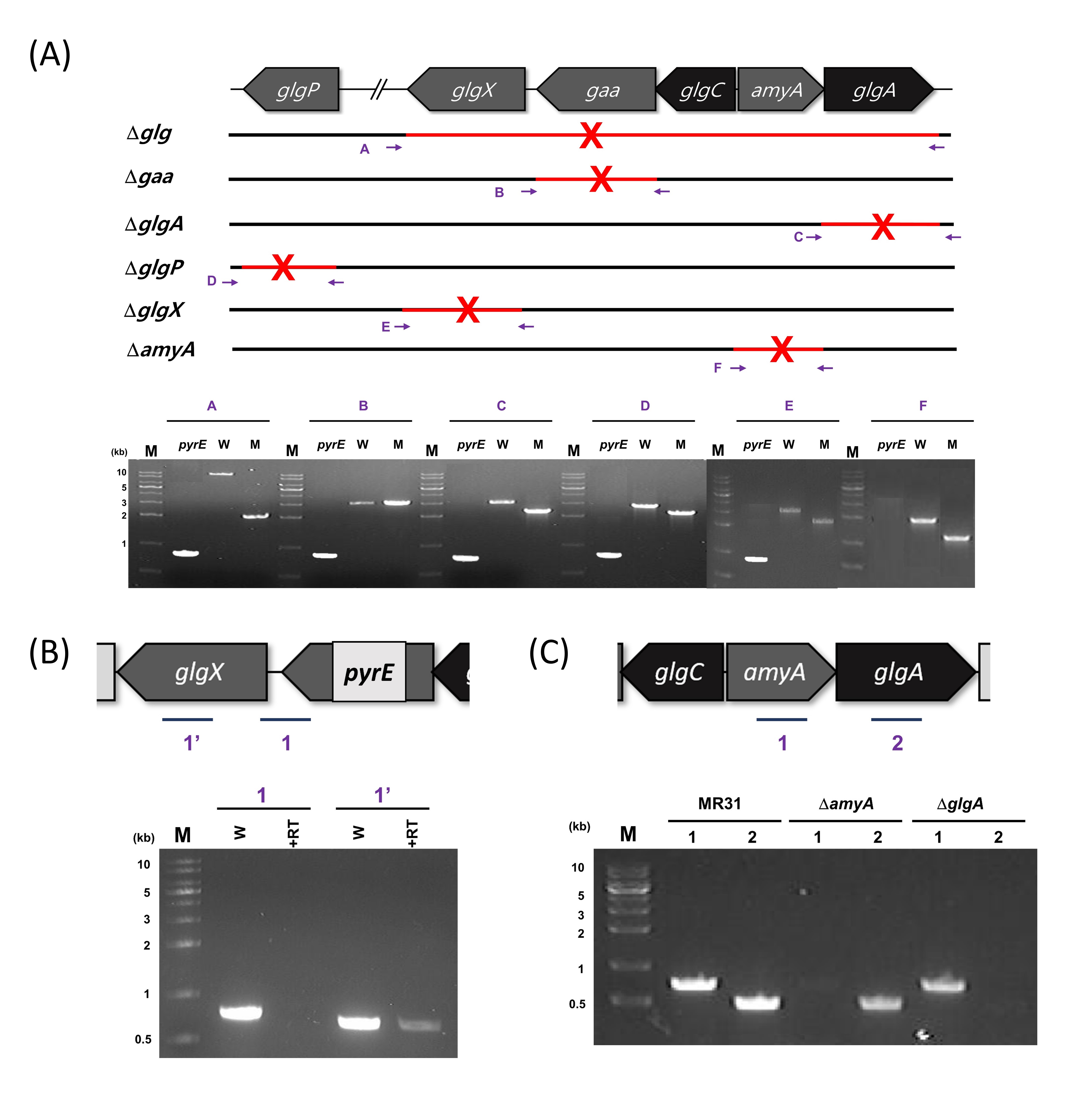

Supplement: Supplementary file 3 [file Image_3.JPEG]

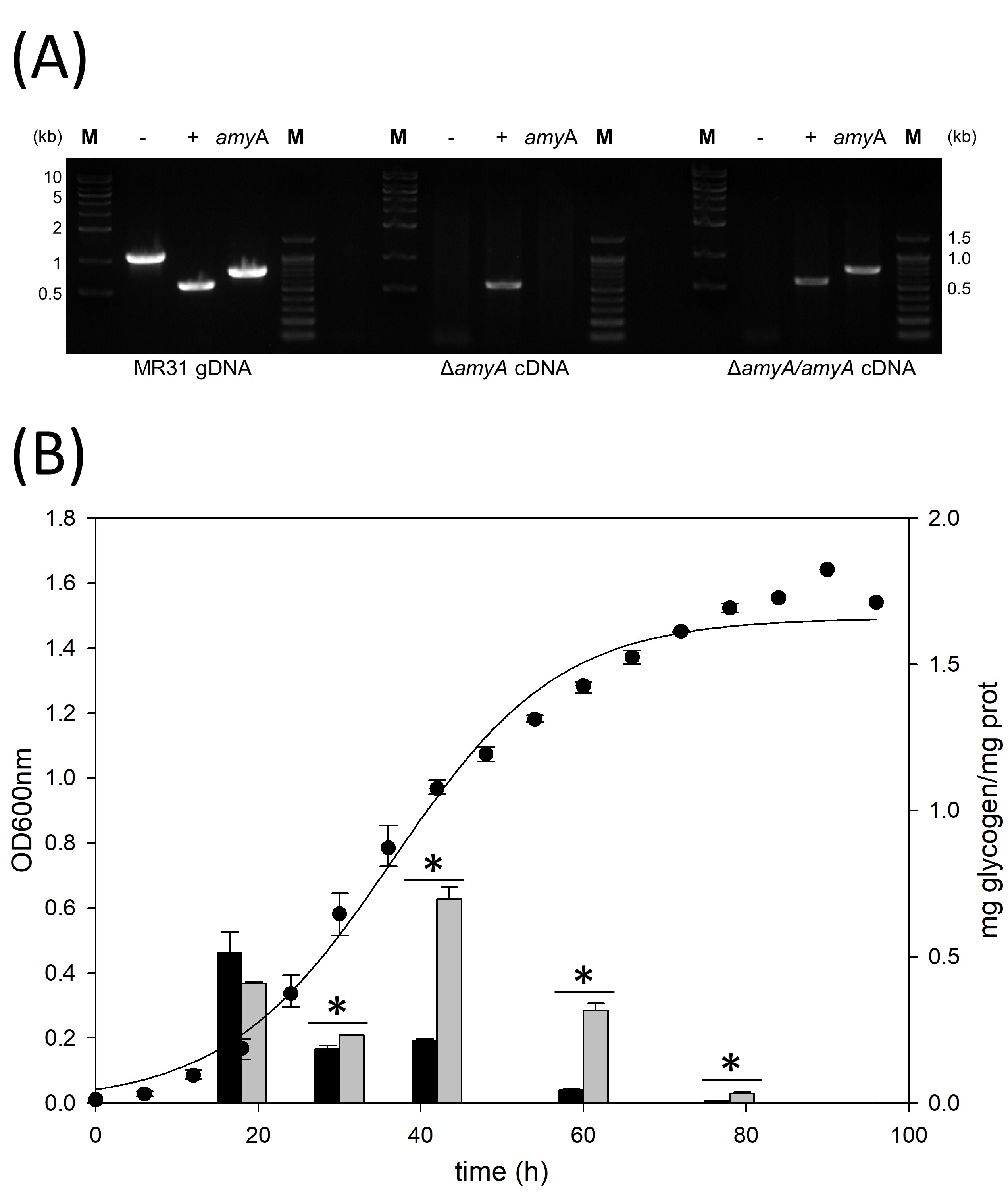

Supplement: Supplementary file 4 [file Image_4.JPEG]

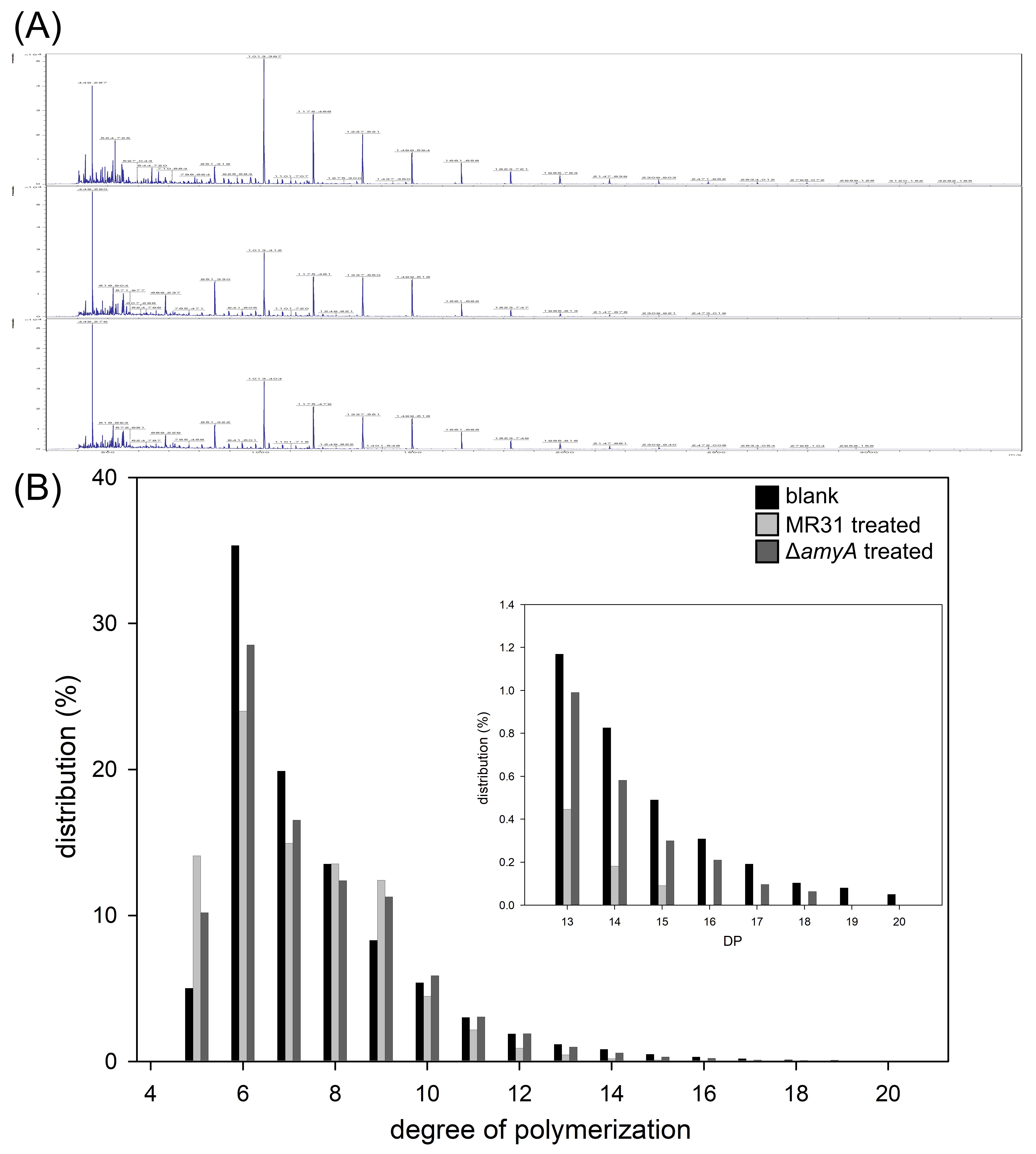

Supplement: Supplementary file 5 [file Image_5.JPEG]

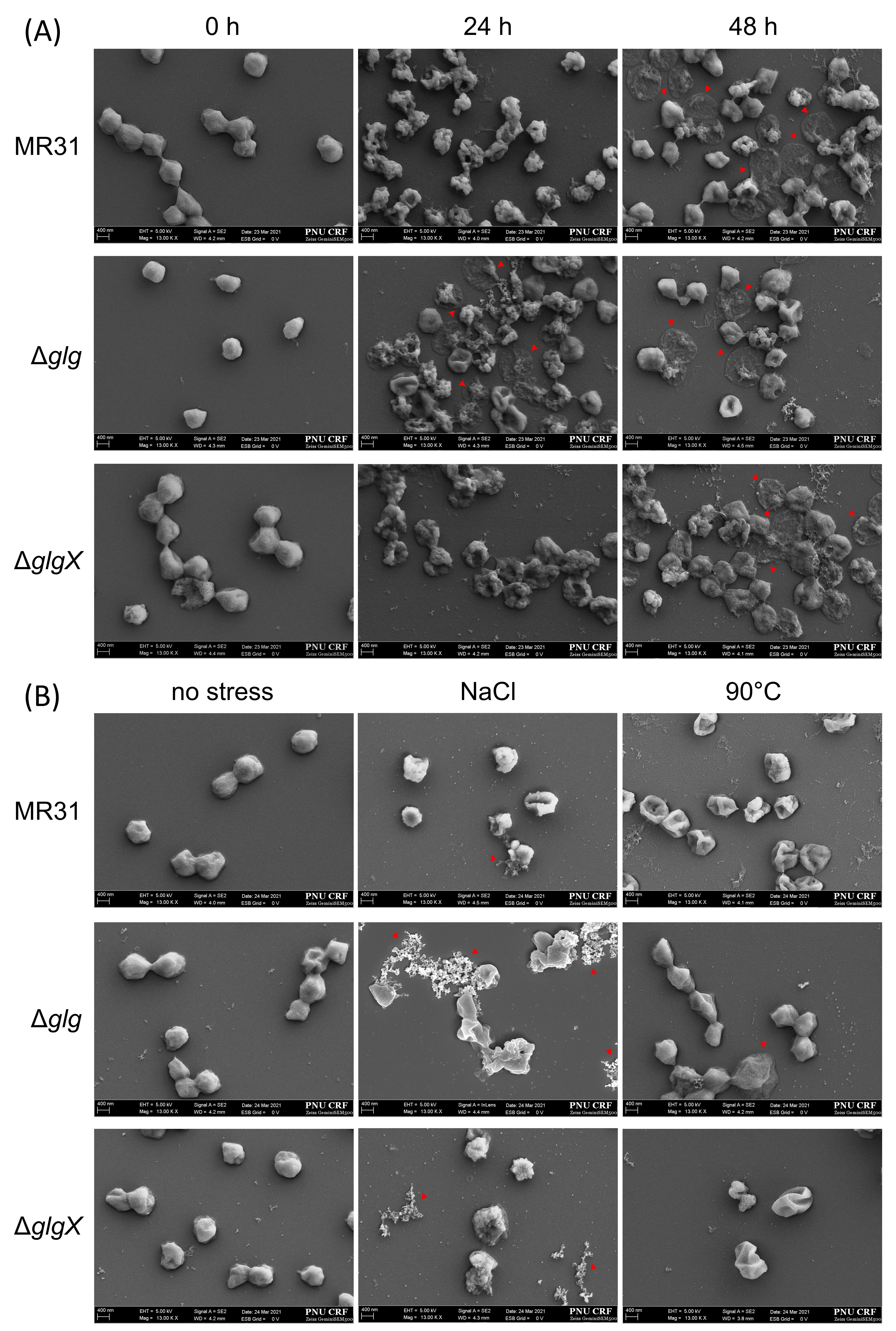

Supplement: Supplementary file 6 [file Image_6.JPEG]
