# Supplementary material for: Identification of the Genes Related to the Glycogen Metabolism in Hyperthermophilic Archaeon, Sulfolobus acidocaldarius
Source: Front Microbiol. 2021 May 13;12:661053. doi: 10.3389/fmicb.2021.661053 (PMC8158581; doi:10.3389/fmicb.2021.661053)
Supplement: Supplementary file 7 [file Data_Sheet_1.docx]

Supplementary Material

Supplementary Table 1. Primers used in this study

| primer | sequence (5'→3') | Remarks |
| --- | --- | --- |
| RT-PCR | | |
| EJ 15 | CGATGGATCCGATGAGATACGTCAGTATTGGTAA | for amplification of intergenic region of *glgC* and*amyA*(for RT-PCR) |
| EJ 16 | GTTACTCGAGAATTGCTTCTTTCATCTGGTGAGA | for amplification of intergenic region of *glgC*and *amyA* (for RT-PCR) |
| EJ 17 | CCTGTATACGCGTATAAGATGGA | for amplification of intergenic region of *gaa-glgC* (for RT-PCR) |
| EJ 18 | GAAGTAGGGATCCAACTGGTTTCCCATTAGTGT | for amplification of intergenic region of *gaa-glgC* (for RT-PCR) |
| EJ 19 | CCAGTTGGATCCCTACTTCCTGAACAAGTTGA | for amplification of intergenic region of *glgX-gaa* (for RT-PCR) |
| EJ 20 | GCGTGAATATAAGGTTATCAGGCTGAG | for amplification of intergenic region of *glgX-gaa* (for RT-PCR) |
| JH 17 | CTTAAAGGATCCCCAGCAGAGGTTCACTCATA | for amplification of intergenic region of *amyA-glgA* (for RT-PCR) |
| JH 18 | CCAAATTTCCATGTTCAAACCCTAGCTTGTA | for amplification of intergenic region of *amyA-glgA* (for RT-PCR) |
| JH 24 | GTCAGCCTTTACAGATACGA GGGTCG | For amplification of intragenic region of *saci_1197* gene (for RT-PCR) |
| JH 25 | ACCCCTGTGAAATAGAATTTCCCCC | For amplification of intragenic region of *saci_1197* gene (for RT-PCR) |
| JH 13 | CTATCGTATACAAGAAAGGTGGAGTTTTTA | For amplification of intragenic region of *saci 1200* gene (for RT-PCR) |
| JH 14 | TGCTGGGGATCCTTTAAGGTACTCTTTACCCAAC | For amplification of intragenic region of *saci 1200* gene (for RT-PCR) |
| JH 38 | TCACTTCTGGCTAGGGCTGT | For amplification of intragenic region of *saci 1201* gene (for RT-PCR) |
| JH 39 | TCCTCAGTCCCAAACTTGCT | For amplification of intragenic region of *saci 1201* gene (for RT-PCR) |
| Mutant construction | | |
| EJ 1 | GATGACTCATGGACTGGTATTGA | For disruption of *glg* operon, specific for downstream of *saci_1196* |
| EJ 2 | TTTCTGCAGGATCCCCCGAGTCACAGTGCAAGTA | For disruption of *glg* operon, specific for downstream of *saci_1196*, introducing *Bam*HI and upstream of *saci_1201* |
| EJ 3 | GACTCGGGGGATCCTGCAGAAAGAAGTTCCACA | For disruption of *glg* operon, specific for downstream of *saci_1201*, introducing *Bam*HI and downstream of *saci_1196* |
| EJ 4 | ATTGCAGCTTCACCAACACA | For disruption of *glg* operon, specific for downstream of *saci_1201* |
| MH 12 | AACTCTCTGATTGGGTACTGTGGTC | For disruption of *glgA* gene, specific for upstream of *saci_1201* |
| MH 13 | ATTCTCATTAGGATCCGTCCCAATCCCCCTAGACTA | For disruption of *glgA* gene, specific for upstream of *saci_1201*, introducing *Bam*HI and downstream of *saci_1201* |
| MH 14 | GGATTGGGACGGATCCTAATGAGAATTTCAGATGGTCAGC | For disruption of *glgA* gene, specific for downstream of *saci_1201*, introducing *Bam*HI and upstream of *saci_1201* |
| MH 15 | AGGCGCAGGAGAACAACAAAT | For disruption of *glgA* gene, specific for downstream of *saci_1201* |
| JH 13 | CTATCGTATACAAGAAAGGTGGAGTTTTTA | For disruption of *amyA*, specific for gene of interest in *saci_1200* |
| JH 14 | TGCTGGGGATCCTTTAAGGTACTCTTTACCCAAC | For disruption of *amyA* specific for gene of interest in *saci_1200*, introducing *Bam*HI and downstream of *saci_1200* |
| JH 15 | AAGTAGCTAGGCTTTATGTGATACCTTGC | For disruption of *amyA* specific for upstream of *saci_1200*, introducing downstream of *sso0615* (*pyrE*) |
| JH 16 | TGAAGGATCCTGATATTGAATAGAATACC | For disruption of *amyA*, specific for upstream of *saci_1200*, introducing *Bam*HI |
| JH 17 | CTTAAAGGATCCCCAGCAGAGGTTCACTCATA | For disruption of *amyA*, specific for downstream of *saci_1200*, introducing *Bam*HI and GOI of *saci_1200* |
| JH 18 | CCAAATTTCCATGTTCAAACCCTAGCTTGTA | For disruption of *amyA*, specific for downstream of *saci_1200* |
| JH 19 | AAAGGGATCCAATGAAACTACTTTCCCTGATAG | For disruption of *amyA*, specific for upstream of *sso0615*, introducing *Bam*HI |
| JH 20 | GCCTAGCTACTTTTCAACATTCTTCACCAAA | For disruption of *amyA*, specific for upstream of *sso0615*, introducing upstream of *saci_1200* |
| JH 9 | AGTAGGTATGT AATACAGATAGTCGG | For disruption of *glgP*, specific for upstream of *saci_0294* |
| JH 10 | CCTTATGGATCCTTATATTTTGATGCCTCTAG | For disruption of *glgP*, specific for upstream of *saci_0294*, introducing *Bam*HI and downstream of *saci_0294* |
| JH 11 | ATATAAGGATCCATAAGGCTGAATCTGATAGA | For disruption of *glgP*, specific for downstream of *saci_0294*, introducing *Bam*HI and upstream of *saci_0294* |
| JH 12 | CTTAATGAGGGCTAGTAAGTAACATTT | For disruption of *glgP*, specific for downstream of *saci_0294* |
| SZ 11 | AGACCCAATAATGGTATCTACTATCAA | For disruption of *glgX* gene, specific for upstream of *saci_1197* |
| SZ 12 | GAATAAGGATCCAATACCACAGATCGTTGAGG | For disruption of *glgX* gene, specific for upstream of *saci_1197*, introducing *Bam*HI and downstream of *saci_1197* |
| SZ 13 | GGTATTGGATCCTTATTCCAACGGTGGAGCTA | For disruption of *glgX* gene, specific for downstream of *saci_1197*, introducing *Bam*HI and upstream of *saci_1197* |
| SZ 14 | CTTAAGTATGTCTGCAGCGGTAATTAC | For disruption of *glgX* gene, specific for downstream of *saci_1197* |
| EJ 17 | CCTGTATACGCGTATAAGATGGA | For disruption of *gaa*, specific for downstream of *saci_1198* |
| EJ 18 | GAAGTAGGGATCCAACTGGTTTCCCATTAGTGT | For disruption of *gaa*, specific for downstream of *saci_1198*, introducing *Bam*HI and upstream of *saci_1198* |
| EJ 19 | CCAGTTGGATCCCTACTTCCTGAACAAGTTGA | For disruption of *gaa*, specific for downstream of *saci_1198*, introducing *Bam*HI and downstream of *saci_1198* |
| EJ 20 | GCGTGAATATAAGGTTATCAGGCTGAG | For disruption of *gaa*, specific for downstream of *saci_1198* |
| EJ 12 | ATCTCCGCGGAATTCCGCAACACCAAACTC | For complementation of *amyA*, specific for upstream region of *saci_1200*, containing *Sac*Ⅱ |
| AR 169 | GTGCTAGTGGTGGTGGTGGTGGTGTTGCTTAATCCACTCTTG | For complementation of *amyA*, specific for downstream region of *saci_1200*, introducing downstream of *saci_0155* |
| KH 44 | CACCACCACCACTAGCACAACTAACAATTTATT | For complementation of *amyA*, specific for downstream region of *saci_0155*, introducing downstream of *saci_1200* |
| KH 42 | TTCTCCGCGGTTCAATTATTCAGATTTA | For complementation of *amyA*, specific for downstream region of *saci_0155*, containing *Sac*Ⅱ |
| Promoter assay | | |
| EJ 25 | CGTCCCGCGGTCCGCACGTTAATTATGTTCC | For amplification of upstream region of *saci_0294* gene, containing *Sac*II |
| EJ 26 | AAATGAGTACGCTATTTCTGGTGTTATTGATATTAT | For amplification of upstream region of *saci_0294* gene, containing *lacS* (*sso3019*) sequence |
| EJ 27 | CCAGAAATAGCGTACTCATTTCCAAATAGCTTTAG | For amplification of *lacS* gene, containing *saci_0294* sequence |
| EJ 9 | AGTACCGCGGCGCTCTCTTTTCCTTCCATT | For amplification of upstream region of *saci_1199* gene, containing *Sac*II |
| EJ 10 | ATGAGTACATATCTTCTATTCTAGTCATCAGTAATA | For amplification of upstream region of *saci_1199* gene, containing *lacS* sequence |
| EJ 11 | ATAGAAGATATGTACTCATTTCCAAATAGCTTTAG | For amplification of *lacS* gene, containing *saci_1199* sequence |
| EJ 12 | ATCTCCGCGGAATTCCGCAACACCAAACTC | For amplification of upstream region of *saci_1200* gene, containing *Sac*II |
| EJ 13 | TGAGTACATGAACCCTAACATGACATTTCTCA | For amplification of upstream region of *saci_1200* gene, containing *lacS* sequence |
| EJ 14 | ATCTTAGGGTTCATGTACTCATTTCCAAATAGCTTTAG | For amplification of *lacS* gene, containing *saci_1200* sequence |
| KH 52 | GAAACCGCGGGCAATCTAATG | For amplification of *lacS* gene, containing *Sac*II |

Restriction sites were underlined.

Supplementary Figure 1. Confirmation of *glg* operon. (A) Arrangement of the genes involved in the glycogen metabolism in *S. acidocaldarius*. Putative promoter regions are presented with red arrow. To determine that *glgC–gaa–glgX* and *amyA–glgA* are co-transcribed, (B) RT–PCR was conducted. The cells were harvested when the OD reached 0.3 and the total RNA was extracted. After the removal of DNA by DNaseI, cDNA was synthesized by reverse transcriptase. The intergenic regions of *glgX–gaa*,*gaa–glgC*, *glgC–amyA*, and *amyA–glgA* were amplified by each primer set by using cDNA as a template. Each region is underlined with numbering. C+, an intragenic region of *gdhA* amplified with cDNA as a positive control (approximately 0.5 kb); W, fragment amplified with genomic DNA; +RT, fragment amplified with cDNA; -RT, fragment amplified with RNA as a negative control.

Supplementary Figure 2. Alignment of putative promoters of *glg* operon. (A) Putative promoter regions of *glgC–gaa–glgX*, *amyA–glgA*, and *glgP* were used to make a sequence logo by WebLogo (http://weblogo.berkeley.edu/). Upstream region ranging -50 to -1 from start codon was used. (B) Putative promoter of *glgX* was aligned to the promoter region of *glgC–gaa–glgX*, *amyA–glgA*, and *glgP*. Red boxes indicate conserved A/T rich sequences.

Supplementary Figure 3. Confirmation of each mutant by PCR and RT-PCR. (A) Red lines represent the deleted region of each gene. M, 1 kb size markers; *pyrE*, PCR products of the mutant gDNA; W, PCR products amplified with specific primer set using MR31 gDNA as template; M, PCR products amplified with specific primers using mutant gDNA as template. MR31 and *gaa* deletion mutant showed similar size of PCR product when *gaa*-specific primers are used, as the size of deleted fragment and the inserted *pyrE* gene was similar. (B) Confirmation of the transcription of *glgX* gene in *gaa* deletion mutant by RT-PCR. (C) In *amyA* deletion mutant, *glgA* was successfully amplified from mRNA, and *amyA* was amplified from *glgA* deletion mutant, suggesting that each mutant did not affect the transcription of the neighboring gene.

Supplementary Figure 4. Measurement of glycogen content in *amyA* complementation strain. (A) Confirmation of *amyA* complementation by RT-PCR. M, 1 kb size marker and 100 bp size marker; -, intergenic region of *saci_2122* and *saci_2123*; +, intragenic region of *saci_2122*; *amyA*, intergenic region of *saci_1200*. Genomic DNA of *S. acidocaldarius* MR31, cDNA of *amyA* deletion strain, and *amyA* complementation strain was used as template. In *amyA* complementation strain, intragenic region of *amyA* was amplified. (B) Growth and glycogen content of *amyA* complementation strain. The cells were inoculated with the initial OD of 0.01 and grown in YT media supplemented with 0.2% glucose. Black and gray bars represent the glycogen content of soluble and membrane-bound fraction, respectively. The significant difference between glycogen content of each fraction at the same time point was represented by asterisks with *p*-value < 0.05.

Supplementary Figure 5. (A) Mass spectra of glycogen branches before and after treatment of MR31 cell-free extract, and Δ*amyA* cell-free extract. Blank means a sample without cell treatment. Mass spectra of each sample were detected ranging from 400–3500 m/z by MALDI-TOF mass spectrometry. (B) Histogram of chain distribution of glycogen branches. Chain distribution of each sample was normalized tointensity = 100%.

Supplementary Figure 6. Morphology of MR31, *glg* deletion strain, and *glgX* deletion strain in stress condition. (A) Cell morphology in nutrient starvation condition. Cells were collected after 0 h, 24 h, and 48 h of incubation in Brock’s basal media. (B) Cell morphology in osmotic stressand heat stress conditions. Cells were inoculated into YT media supplemented with 0.2% glucose and 300 mM of NaCl to expose cells in osmotic stress. For heat stress condition, cells were grown in YT media supplemented with 0.2% glucose at 90°C. Cells were used after 14 h of incubation in each condition. Red arrows represent the cells with changed morphology.
